# Dependent Competing Failure Processes in Reliability Systems

**DOI:** 10.3390/e26060444

**Published:** 2024-05-25

**Authors:** Jewgeni H. Dshalalow, Hend Aljahani, Ryan T. White

**Affiliations:** Department of Mathematical Science, College of Engineering and Science, Florida Institute of Technology, Melbourne, FL 32901, USArwhite@fit.edu (R.T.W.)

**Keywords:** competing failure processes, extreme shocks, N-critical shocks system, multiple *δ*-shocks, random walk, fluctuation theory, discrete operational calculus, failure time, prefailure time, reliability function, closed form, 90B22, 60K10, 60G50, 60G51, 62N05

## Abstract

This paper deals with a reliability system hit by three types of shocks ranked as harmless, critical, or extreme, depending on their magnitudes, being below $H_{1}$, between $H_{1}$ and $H_{2}$, and above $H_{2}$, respectively. The system’s failure is caused by a single extreme shock or by a total of *N* critical shocks. In addition, the system fails under occurrences of *M* pairs of shocks with lags less than some δ (δ-shocks) in any order. Thus, the system fails when one of the three named cumulative damages occurs first. Thus, it fails due to the competition of the three associated shock processes. We obtain a closed-form joint distribution of the time-to-failure, shock count upon failure, δ-shock count, and cumulative damage to the system on failure, to name a few. In particular, the reliability function directly follows from the marginal distribution of the failure time. In a modified system, we restrict δ-shocks to those with small lags between consecutive harmful shocks. We treat the system as a generalized random walk process and use an embellished variant of discrete operational calculus developed in our earlier work. We demonstrate analytical tractability of our formulas which are also validated, through Monte Carlo simulation.

## 1. Introduction

### 1.1. Competing Failure Processes

The term “competing failure processes” applies to systems periodically or continuously damaged by at least two factors. For example, a system can be hit by shocks of different magnitudes, so that one single extreme shock of a magnitude exceeding a threshold *H* can knock the system down. Or if two consecutive shocks land in the system within a very short period of time, say, with a time lag smaller than a δ, this can ruin the system as well. The second of two such shocks is referred to as a δ-shock. In this simple situation, the system fails when it is hit by an extreme shock or by a δ-shock, whichever of the two comes first. The time of the system’s failure is referred to as the time-to-failure or lifetime of the system. Even though there is one single shock process, we deal with two different types of damages inflicted on the system.

More formally, suppose that the shocks land in the system at times $\tau_{1},\tau_{2},\ldots$ with respective magnitudes $W_{1},W_{2},\ldots$. Thus, a shock at $\tau_{k}$ is extreme when $W_{k}>H,$ and the *k*th shock is a δ-shock if $\tau_{k}-\tau_{k-1}<\delta$. The system fails at time $\tau_{k}$ if the *k*th shock is extreme or it is a δ-shock. In this case, $\tau_{k}$ is the lifetime of the system.

More rigorously, let ν= inf$\left\{n=1,2,\ldots :W_{n}>H\right\}$ and μ= inf$\{m=1,2,\ldots :\tau_{m}-$$\tau_{m-1}<\delta \},$ and ρ=μ∧ν. Then, $\tau_{\rho }$ is the time-to-failure.

Now, we can identify two processes, $R_{1}=\left(\tau_{k},W_{k}:k=0,1,\ldots \right)$ and $R_{2}=(\tau_{k}-$$\tau_{k-1}:k=1,2,\ldots )$, which compete with each other in the sense that $R_{1}$ wins if ν<μ. $R_{2}$ wins if μ<ν, and no process wins when μ=ν. Consequently, we can say that $R_{1}$ and $R_{2}$ compete with each other, and because they are obviously dependent, $R_{1}$ and $R_{2}$ are dependent competing failure processes (or DCFP).

Other examples of DCFP include the degradation (aging) process that is represented by a monotone-increasing function or monotone-increasing or nondecreasing stochastic process φ that runs until it crosses some fixed sustainability threshold *D*. If *T* (whether random or deterministic) is the crossing (first passage) time, then the system fails. Degradation can be accelerated by soft shocks that more quickly degrade the system, causing φ to cross *D* sooner, which may also occur upon the landing of one of the soft shocks that turns out to be fatal. Thus, the combination of the natural degradation and external soft shocks can represent process $R_{1}$. Other shocks can also hit the system as described above as process $R_{1}$, now $R_{2},$ with some shocks being extreme. If the crossing of *D* occurs first and thus causes a so-called soft failure of the system, then $R_{1}$ wins. If an extreme shock (with magnitude W>H) hits the system first and causes a so-called hard failure, $R_{2}$ wins. Altogether, the system fails at some time $\tau_{\rho },$ which is *T* or some time $\tau_{k},$ when an extreme shock occurs.

Note that in the general case, the magnitude $W_{k}$ depends on $\tau_{k}-\tau_{k-1},$ for all $k=1,2,\ldots \;\left(\tau_{0}=0\right)$. Such a process $\left(\tau_{k},W_{k}:k=1,2,\ldots \right)$ is called position-dependent or the process with position-dependent marking. The latter adds yet additional dependence between $R_{1}$ and $R_{2}$.

### 1.2. The System under Study

Consider a reliability system periodically hit by random hard shocks of magnitudes $W_{1},W_{2},\ldots$ taking place at respective times $\tau_{1},\tau_{2},\ldots$. Some of these shocks are harmless, some are critical, and some can be singly fatal (usually called extreme). All shocks are classified by one of the three types dependent on their magnitudes relative to two fixed critical thresholds, $H_{1}<H_{2}$. The harmless shocks are those whose magnitudes $W_{k}$s $\leq H_{1}$. Shocks with magnitudes $W_{k}\in \left(H_{1},H_{2}\right]$ are critical, and any single shock of magnitude $W_{k}>H_{2}$ is extreme and thus fatal. The system fails instantly after being hit by a single extreme shock. However, there are *N* critical shocks to be landed in any order that need to knock the system down. The last, *N*th critical, shock is fatal. In a nutshell, a shock is fatal if it is extreme or *N*th critical.

Altogether, the system fails whenever it is being hit by *N* critical shocks or by one extreme shock, whichever of the two events comes first. Note that regarding the critical shocks, it is not a run system, in which critical shocks must follow one another. In our case, the assumptions are looser, allowing the critical shocks to be mixed with harmless shocks that cause failure only when their total number reaches *N*.

Further embellished, the system is refined in such a way that the harmless shocks are not that harmless after all. Namely, the system can also be fatally harmed if any two consecutive shocks (including those categorized as harmless) land with a time lag less than some δ>0. The second shock is referred to as a δ-shock. Now we have three different forces that can trigger system’s failure:

(*i*) A total of *N* critical shocks.
(*ii*) One extreme shock.
(*iii*) Two consecutive shocks, with a time lag between them less than δ.
  (*iii′*) An embellished variant of $\left(iii\right)$ is due to the system’s policy with a total of *M* δ-shocks. Note that *M* δ-shocks apply to multiple δ-shocks occurring in any order, even consecutively. For example, if *M* δ-shocks are consecutive, starting, say, at $\tau_{i+1},$ the i+1st shock (deemed as the first δ-shock) lands within a period of time less than δ counted from the *i*th shock at $\tau_{i},$ followed by i+2nd shock at $\tau_{i+2}$ with a time lag less than δ from $\tau_{i+1}$, *…*, followed by i+Mth shock at $\tau_{i+M}$ with a time lag less than δ from $\tau_{i+M-1}$. An *M*-δ-shock model in which δ-shocks occur consecutively is called a δ-run model.

The system fails at some $\tau_{\rho }\in \left\{\tau_{1},\tau_{2},\ldots \right\}$ if at least one of the three events $\left(i–iii\right)$ or $\left(i,ii\right)$ with $\left(iii^{\prime }\right)$ above takes place.

Related Literature. For convenience, we break the entirety of the literature into four subsections whose contents may occasionally overlap.

### 1.3. DCFP

The systems with DCFP are more complex than those introduced in Section 1.1. Most work is focused on computing the reliability function $R\left(t\right)=P\left\{\tau_{\rho }>t\right\},t\geq 0$ and the utility of the total probability formula to arrive at $R\left(t\right)$ that typically includes one or multiple series and integrals, with numerical results or Monte Carlo simulation, all used to compute special cases. Many such papers include interesting practical examples of complex devices in engineering and computer science where such DCFP take place and with the need to proceed with an associated probabilistic analysis.

For example, Che et al. [1] in 2018 studied a system with degradation driven by a monotone-increasing stochastic process intertwined with occasional soft shocks entering the system according to a marked Poisson process. That same process of soft shocks also hits other components but with different impacts, and they are referred to as hard shocks, some of which are extreme due to their magnitudes. The first such extreme shock knocks the system down unless the system fails earlier due to a combination of degradation and soft shocks.

A somewhat similar system was studied in 2018 by Zhang et al. [2], in 2017 by Hao et al. [3], in 2023 by Feng et al. [4] (where degradation is modeled by a gamma process), and in 2021 by Bian et al. [5], who dealt with a multicomponent system. In 2021, Sun et al. [6] studied yet another similar system, where, however, the degradation process is modeled by drifted Brownian motion (which is nonmonotone).

Now, Hao and Yang [7] in 2018 embellished Hao et al. [3], which they coauthored, by introducing hard failure thresholds and also adding a δ-shock policy to the competition.

An interesting modification of the above was proposed by Liu et al. [8] in 2017, in which the degradation process is rendered nonmonotone to attribute to downhill directions as a self-healing mechanism.

In 2021, Lyu et al. [9] added the third competing process pertaining to the run shock policy. This condition renders the system fail when the magnitudes of *k* consecutive shocks exceed a critical threshold. Furthermore, when the total number of shocks attains a certain value, the degradation rate of soft failure changes. Furthermore, the shocks’ interarrival times follow a phase distribution.

In 2022, Hao and Li [10] investigated DCFP applied to a single-component model, series, parallel, and mixed series and parallel models.

In 2019, Ranjkesh et al. [11] studied a DCFP system where a shock process is Poisson with position-dependent marking. In this system, there is no other degradation process besides the shocks that accumulate until their cumulative damage crosses a fixed threshold. Another competing process is forged using the δ-shock principle. The authors approximate the system’s reliability function.

In 2023, Dshalalow and Aljahani [12] studied an N-critical shock model competing with an aging process.

N-Critical Shocks Models. As a DCFP with multiple processes involved, an N-critical model, along with aging and soft shocks, was studied in 2022 by Dshalalow and White [13]. The aging process was defined as linear with a deterministic slope, and it was combined with soft shocks that accelerated aging, and such a cumulative aging process sooner or later crossed a sustainability threshold. The projection of such a crossing point was the soft failure. After this random point, say η, the system was deemed inoperational and shut off. The system could also fail if it was hit by one of the critical shocks, namely, by the *N*th critical shock, say at the instant $\tau_{\nu }$. Thus, the system fails at time $\eta \wedge \tau_{\nu }$.

In 2012, Jiang et al. [14] studied a variant of such a system with aging, soft shocks (cumulative shocks model), and hard shocks. There are three thresholds, $H_{0}<H_{1}<H_{2},$ of which $H_{2}$ is “critical”. It takes just one shock of a magnitude above $H_{2}$ to knock the system down. However, once *N* shocks cross $H_{0}$ (but not $H_{2}$), the threshold $H_{2}$ is downgraded to $H_{1},$ so that it now takes one $H_{1}$-critical shock (that is, of a lesser magnitude) to knock the system down. Meanwhile, aging, along with soft shocks, takes its course, and if the aging curve crosses some D, the system soft-fails, unless it fails earlier due to an extreme shock. Now we see that while this system is not exactly N-critical shock, it carries some elements of the N-critical shock protocol.

An N-critical shock system was studied earlier by Cha and Finkelstein [15] in 2011, but with no aging. Wu et al. [16] in 2022 also studied an N-critical shock system with no aging under the assumption that shocks arrive according to a Markov renewal process.

Most recently, an N-critical shock system appeared in 2023 in works by Wei et al. [17] and Dshalalow and Aljahani [12]. The authors of [17] also included shock-dependent maintenance. In [12], Dshalalow and Aljahani worked with an aging process driven by a nonspecified monotone-increasing function δ that crosses a threshold *D* at point $T=\delta^{-1}\left(D\right)$ that can be observed only with some random delay, that is, at some epoch of time when the system’s failure can be verified. The system can fail earlier if it is hit by a total of *N* critical shocks. So, there is a combination of DCFP and N-critical shocks in one system. The authors of [12] arrive at closed-form functionals representing the joint distribution of the lifetime of the system, the overall damage to the system upon failure, and other characteristics, such as prefailure time and the associated damage.

Run Shock Models. A run shock system is a special case of an N-critical shock system, compared to which, a run shock requires *N* consecutive shocks to occur to knock down the system, whereas an N-critical shock policy allows *N* critical shocks to mix with noncritical shocks in any order. Furthermore, any consecutive occurrence of *N* critical shocks is not excluded from the N-critical shock protocol, and thus formally, the N-critical policy is more relaxed compared to the run shock policy. For that reason alone, the N-critical policy seems to apply to a wider class of reliability systems.

Here is another shortcoming of the run shock policy. Suppose a system is hit by a run of N−1 consecutive critical shocks followed by one noncritical shock, then followed by another run of N−1 consecutive critical shocks and one noncritical shock, and so on. It seems likely that it takes a while (if ever) to come up against a run of *N*-critical shocks before the system becomes “inoperational” as per the run shock nomenclature. It appears that in this situation, the system may become exhausted much earlier than at an assumed failure time in the run shock framework.

Yet, run shock represents much earlier modeling with an interesting analytics. An argument for run shocks was given in Mallor and Omey [18] in 2001 that if applied to insurance claims, only a series of *N* consecutive claims large enough would raise flags. We think that *N* large claims in any order are sufficiently concerning and more realistic. Note that Mallor and Omey [18] were the first to introduce such systems; they also studied such a system in 2003 [19]. Various embellishments of run shock models were studied in Gong et al. [20] in 2018, Eryilmaz and Tekin [21] in 2019, Lyu et al. [9] in 2021, and in 2022 by Wen et al. [22]. Poursaeed [23] in 2021 studied a fairly complex multistate run shock system with different lengths of runs and different categories of failures.

δ-Shock Models. Related to our system are also δ-shock models. As already mentioned in the description of our model, the failure of the system is stipulated by the first occurrence of two consecutive shocks with a time lag of less than some fixed δ. This policy pertains to our model when M=1. The plain δ-shock policy is often implemented whenever shock damages (or magnitudes) are hard to observe. A δ-shock model was first introduced by Li et al. [24] in 1999 followed by Li and Kong [25] in 2007 under the same assumptions, targeting the asymptotic behavior of the system when δ→0. Another plain δ-shock model from the same period was analyzed by Tang and Lam [26] in 2006.

Embellishments of δ-policy are seen in later works, like one in the article by Parvardeh and Balakrishnan [27], dated 2015. Here, the system is deemed to fail when $\left(a\right)$ there is an occurrence of one δ-shock, or $\left(b\right)$ the magnitude of any single extreme shock is larger than some H, whichever comes first. Eryilmaz [28] combined run shocks and *M* δ-shocks $\left(\mathrm{that}\;\mathrm{is},\;a\;\mathrm{run}\;\mathrm{and}\;\delta \text{-}\mathrm{run}\;\mathrm{model}\;\mathrm{in}\;\mathrm{one}\right)$, which was a significant upgrade of [27] even though the paper by Eryilmaz appeared three years earlier, in 2012, compared to [27].

An interesting embellishment of Eryilmaz’s δ-run model [28] was introduced by Jiang [29] in 2020. Such a system had *N* different failure thresholds $\delta_{1}>\delta_{2}>\ldots >\delta_{N}>0$. If the time lag between two consecutive shocks lies in $\left(\delta_{i+1},\delta_{i}\right),i=1,\ldots ,N$ ($\delta_{N+1}=0$), the system is associated with *i*th failure type. The *N*th type is irreparable and the whole system needs replacement, while the first N−1 types allow repair.

Other various embellishments of δ-shock systems were studied by Lorv et al. [30] in 2020, Wu et al. [31] in 2022, Roozegar et al. [32] in 2023, Doostmoradi [33] in 2023, and Lyu et al. [34] also in 2023.

**Remark** **1** (Some applications). *While extreme shocks naturally occur in numerous real-world situations and the reliability literature, δ-shock systems are slightly less popular, while N-critical models are especially rare. Yet such situations often arise in connection with various insurance claims or a combination of claims, citations, and violations. It is particularly apparent with car insurance. Each insured automobile driver knows that every incident, even the one caused by another driver, triggers an unwanted citation, collectively crossing a specific threshold ending in cancellation of a policy, because the driver is deemed to pose a risk for the underwriter. Not every accident or incident (i.e., shock) is equal (shock’s magnitudes), but roughly a certain number of incidents deemed critical can cause a policy’s cancellation. The time lag between such shocks can also play a major role. Typically, several incidents mixed with traffic violations occurring within short time intervals carry a higher risk of cancellation than the same amount of such incidents spread over a longer period.*

*δ-shocks that occur in technology or electronic devices are regarded more hazardous because they significantly reduce the chance for the system to partially recover after being hit by harmful shocks. Consider, for example, a car suspension system that is periodically hit by bumps or holes. One such critical hit may require a small amount of maintenance. Yet even with maintenance, there is a limit to how many such hits the suspension can sustain before having to undergo a complete and costly replacement. Such hits become even more dangerous if they occur within time intervals short enough without giving the system an opportunity for partial maintenance.*

*The same applies to biological organisms like human bodies periodically traumatized by various diseases that wear out our immune system. Those ailments occurring with shorter lags reduce the odds for our bodies to (even partially) recover, and thus, such shocks become more life-threatening. One of the reasons why δ-shocks are alarming is because after each disorder (harmful shock), our immune system works hard on the body’s recovery, whereas consecutive δ-shocks force the system to multitask.*

*Critical and δ-shocks often take place in the stock market. Any adverse action, such as proposing a controversial budget in Government chambers deemed harmful to the market or raising crude oil prices, can cause the market to stumble. Raising interest rates due to inflation, wars and the expectation of wars, or bad reports about major companies or sectors, to name a few, can be thought of as critical shocks for the market. On the other hand, an economic shock is harmless if it is just noisy and can be easily identified by using special mathematical tools. However, adverse economic or political events can have big impacts on market health, especially if they occur within short time periods, giving the market no opportunity to recover and increasing the risk of a serious crash.*

Our Work. The system under study includes two models. In Model 1, we consider a random process of shocks that are categorized under four types: harmless shocks, critical shocks (with a total of *N* to ruin the system), extreme shocks of which only one is sufficient to knock the system down, and δ-shocks (when two consecutive shocks of any category hit the system within a time interval shorter than δ). We obtain a closed form of joint distribution of the failure time $\tau_{\rho },$ the shock count ρ upon the failure, the cumulative damage to the system upon the failure, and some other useful random characteristics, such as prefailure time $\tau_{\rho -1}$ and the status of the system at $\tau_{\rho -1}$. In particular, it gives the reliability function that directly follows from the marginal distribution of $\tau_{\rho }$.

In Model 2, we define a δ-shock as a consecutive critical shock. Consequently, the harmless shocks are excluded, or rather, bypassed. Thus, if two critical shocks, say, at $T_{j}$ and $T_{j+1},$ hit the system, one after another, the time lag between them must be smaller than δ. There can be some or many harmless shocks enclosed between $T_{j}$ and $T_{j+1}$ landing at times $\tau_{s},\tau_{s+1},\ldots$, but they are not counted as a threat to the system, even though their time lags are even shorter. Note that a shock at $T_{j+1}$, instead of being critical, can also be extreme and thus counted as a δ-shock. Then for Model 2, we obtain similar characteristics.

Because we treat the system as a generalized random walk process and use an embellished variant of discrete operational calculus, our techniques differ from all others in the reliability literature. We demonstrate analytical tractability of the results obtained from a number of special cases and marginal distributions, leading to compact and explicit expressions, and we discuss various examples. Furthermore, we validate our results by Monte Carlo simulation.

## 2. Formalism of Model 1

The current section deals with modeling of a reliability system referred to as Model 1. Section 3 deals with formulas for the joint distribution of key characteristics of the shock process, including the prediction of the time-to-failure established in Theorem 1 (using fluctuation analysis of random walk processes) that by far exceeds what Model 1 originally targets. Section 4, Section 5 and Section 6 continue with Model 1, discussing various applications and special cases and validating the results associated with Theorem 1 by Monte Carlo simulation. Section 7, Section 8, Section 9 and Section 10 deal with Model 2, which emerged from Model 1.

Let $\left[W\right]$ denote the equivalent class of all stochastically equivalent r.v.s on a given probability space $\left(\Omega ,F,P\right)$ valued in $R_{+}$ such that $W\in \left[W\right]$ represents the magnitude of some shock. Then, the sample space Ω can be partitioned into the three events:

(1) $$E_{1}=\left\{W\leq H_{1}\right\},E_{2}=\left\{H_{1}<W\leq H_{2}\right\},E_{3}=\left\{H_{2}<W\right\}.$$

Let

(2) $$Y=1_{E_{2}}+N1_{E_{3}}$$

with distribution

(3) $$a=P\left(E_{3}\right),b=P\left(E_{2}\right),c=P\left(E_{1}\right).$$

Here $1_{A}$ stands for the indicator function parametrized by a fixed set *A* that can be an event like in (2).

Note that the way r.v. *Y* is defined implies that one extreme shock that ruins the system at once has the strength of *N* critical shocks. Furthermore, it takes *N* critical shocks to makes the system inoperational. That said, with a sequence $Y_{1},Y_{2},\ldots ,$ the system is immune to harmless shocks, that is, when respective *Y*’s equal 0 (with probability *c*). With $Y_{k_{1}}=Y_{k_{2}}=\ldots =Y_{k_{N}}=1,$ the system is stricken by a total of *N* critical shocks of which the fatal one lands at $\tau_{k_{N}}$. The system can be ruined earlier at some $\tau_{n}$ if $Y_{n}=N$, corresponding to the first and only extreme shock.

Obviously, $\left\{Y=N\right\}=E_{3},\left\{Y=1\right\}=E_{2},$ and $\left\{Y=0\right\}=E_{1}$. Then the probability generating function (pgf) of *Y* is

(4) $$Ez^{Y}=\int z^{Y}dP=\int z^{y}P_{Y}\left(dy\right)=\sum_{y}z^{y}P\left\{Y=y\right\}=az^{N}+bz+c.$$

As mentioned, the impact of one extreme shock is equivalent to *N* critical shocks that occur in any order and are mixed with harmless shocks, in particular, when N=1, $Y=1_{E_{2}\cup E_{3}}$, eliminating the need for two thresholds $H_{1}$ and $H_{2}$ and making any critical shock equally extreme. The pgf of *Y* reduces to $Ez^{Y}=\left(a+b\right)z+c,$ making *Y* a Bernoulli r.v. with events $E_{2}$ and $E_{3}$ merged. On the other hand, when *N* becomes very large, it seems like one extreme shock is a more likely scenario of system’s failure, although the latter also depends on *a* and *b*.

Suppose $\left(\tau_{k}:k=1,2,\ldots \right)$ is a point process on $R_{+},$ that is, $\sum_{k=1}^{\infty }\varepsilon_{\tau_{k}}$$\left(\varepsilon_{a}\;\mathrm{is}\;a\;\mathrm{point}\;\mathrm{mass}\right)$ a random measure, representing the times when the shocks hit the system, such that almost surely (a.s.) $\tau_{k}\to \infty$ as k→∞.

Let $\left(W_{k}:k=1,2,\ldots \right)\subseteq \left[W\right]$ be a sequence of iid (independent and identically distributed) r.v.s representing magnitudes of shocks exerted on the system at respective times $\left(\tau_{k}\right)$. The process of the shocks’ times and magnitudes could be specified by the marked point process. However, if we want to easily distinguish the impacts of the shocks as per (1)–(4), we would rather turn to the auxiliary sequence $\left(Y_{k}\right)$ associated with $\left(W_{k}\right)$, specified in (2), and utilize the marked point process $\sum_{k=1}^{\infty }Y_{k}\varepsilon_{\tau_{k}}$. Note that while the r.v. $Y_{k}$ is closely related to $W_{k},$ it does not reveal the magnitude of $W_{k}$ other than pointing out what category the shock with magnitude $W_{k}$ belongs to. But the sequence $\left\{Y_{k}\right\}$ carries enough information on $\left\{W_{k}\right\}$ to lay the foundation for our forthcoming analysis of a discrete-valued random walk that we are going to employ throughout this paper, and thus it well serves its purpose.

To proceed further, we have

(5) $$B_{n}=\sum_{k=1}^{n}Y_{k},\;n=1,2,\ldots ,$$

and we form the associated sequence of partial sums of $\left\{Y_{k}\right\}$ and define

(6) $$\nu =min\left\{n:B_{n}\geq N\right\}.$$

where ν is the ruin index (also the nominal count of harmful hits) exerted upon the system in the absence of any other formal cause of the system’s failure.

We note that there is a situation when $B_{\nu }$ is strictly greater than *N*. It occurs when $B_{\nu -1}<N$ and $Y_{\nu }=N,$ that is, when $W_{\nu }>H_{2}$. Thus, the system has accumulated $B_{\nu -1}$ critical shocks (short of *N*), and a shock at $\tau_{\nu }$ turns out to be extreme (valued equivalent to *N* critical shocks), implying that $B_{\nu }=B_{\nu -1}+N$, which can be strictly greater than *N*.

Observe that even if *N* is large, the system still can fail fairly soon, because one extreme shock carries *N* and it knocks the system down on its first occurrence, while a sequence of critical shocks gets spread out over time if probability *b* of a critical shock is small enough compared to *a*, making *N* such shocks unlikely to occur soon. So, the competition between critical and extreme shocks is more flexible compared to a system under critical shocks alone, and it is driven by *N* as well as *a* and *b*.

Furthermore, the system can also go under if there are *M* instances (in any order) when the time lag between any two consecutive (even harmless) shocks is less than some (small) real number δ. This is formalized as follows. Let $X_{i}\in \left[X\right]$ such that

(7) $$X_{i}=1_{\left\{\left|\left(\tau_{i-1},\tau_{i}\right)\right|<\delta \right\}},i=1,2,\ldots ,\tau_{0}=0,$$

assuming that

(8) $$\left|\left(\tau_{i-1},\tau_{i}\right)\right|\in \left[\Delta \right],i=1,2,\ldots ,\tau_{0}=0,\left|\cdot \right|\mathrm{is}\;\;\mathrm{the}\;\;\;\mathrm{Lebesgue}\;\;\mathrm{measure}$$

(later on, we discuss other options for $\tau_{0}$), that is, the lengths $\Delta_{i}$s of intervals $\left(\tau_{i-1},\tau_{i}\right)$s are identically distributed as some r.v. Δ.

The r.v. $X_{i}$ is Bernoulli with the marginal pgf

(9) $$Ey^{X_{i}}=\alpha y+\beta ,\;$$

where

(10) $$\alpha =P\left\{\Delta <\delta \right\}.$$

Thus, $\left\{X_{i}\right\}$ is the sequence of i.i.d. Bernoulli r.v.s counting δ-shocks.

We would like to call $X_{i}$s and $Y_{i}$s the shocks identifiers.

**Remark** **2.** *From this on, it makes sense to define $X_{0}=Y_{0}=0$ a.s.; while it is clear why we set $X_{0}=0$ (because with M=1, the condition $X_{0}=1$ would make the system instantly fail and bar it from any further development, which makes sense to avoid), with $Y_{0}=0$ we agree to have the system started with one harmless shock if we take into consideration Equations (2) and (3) where $Y\in \left\{0,1,N\right\}$ with the distribution $\left\{c,b,a\right\}$. Of course, we can replace the very rigid and impractical condition $X_{0}=1$ a.s. with $P\left\{X_{0}=1\right\}=q$ instead.*

*Concerning $\left\{Y_{k}\right\},$ we set $Y_{1},Y_{2},\ldots \in \left[Y\right]$. However, we define $Y_{0}=0$ with probability 1, that is, under the assumption that a=b=0 and c=1, which agrees with (2) and (3), and have $Y_{0}$ distributed differently from the rest of $Y_{i}$s. Consequently, the process $\left\{\left(X_{i},Y_{i}\right):i\in N_{0}\right\}$ is delayed renewal.*

*A benefit of such a setting is that $X_{1}$ is a *δ*-shock if $\left|\left(\tau_{0},\tau_{1}\right)\right|<\delta ,$ because with a harmless shock at $\tau_{0},$ we ensure that $X_{1}=1$, in full agreement with its definition in (7) rather than with a conflicting message about $X_{1}$ being 1 according to (7) if there is no shock at all at $\tau_{0}$. Furthermore, it is also in agreement with a forthcoming formula in Corollary 1.*

*We also note that with $Y_{0}=0,$ a harmless shock allegedly at $\tau_{0}$ need not occur exactly at time $\tau_{0},$ but at any time prior to $\tau_{0}$ allowing us to keep a harmless shock on record and assign it to time $\tau_{0}$.*

The joint distribution of $\left(X,\Delta \right)$ is naturally obtained through

(11) $$g\left(y,\theta \right)=Ey^{X}e^{-\theta \Delta }=Ey^{1_{\left\{\Delta <\delta \right\}}}e^{-\theta \Delta }=\alpha \left(\theta \right)y+\beta \left(\theta \right),$$

where

(12) $$\alpha \left(\theta \right)=E1_{\left\{\Delta <\delta \right\}}e^{-\theta \Delta }\;\mathrm{and}\;\beta \left(\theta \right)=E1_{\left\{\Delta \geq \delta \right\}}e^{-\theta \Delta }$$

and

(13) $$\alpha =\alpha \left(0\right)=P\left\{\Delta <\delta \right\},\beta =\beta \left(0\right),\alpha +\beta =1,$$

whereas the marginal LST (Laplace–Stieltjes transform) of Δ is

(14) $$\gamma \left(\theta \right)=Ee^{-\theta \Delta }=g\left(1,\theta \right)=\alpha \left(\theta \right)+\beta \left(\theta \right)\;\mathrm{assuming}\;\frac{1}{\gamma }=E\Delta <\infty .$$

As for the common joint transform $\gamma \left(y,z,\theta \right)=Ey^{X}z^{Y}e^{-\theta \Delta }$ of the sequence $\left(X_{i},Y_{i},\Delta_{i}\right),\;i=1,2,\ldots$, we assume that *Y* is independent of $\left(X,\Delta \right)$. That is, in the context of the marked point process $\left(X_{i},Y_{i},\Delta_{i}\right),i=1,2,\ldots ,\;$ we assume position independent marking. This means that the magnitude $W_{i}$ of the *i*th shock at $\tau_{i}$ (and thus $Y_{i})$ is independent of $\Delta_{i}=\left|\left(\tau_{i-1},\tau_{i}\right)\right|,$ as a common assumption in many real-world reliability systems. Note, however, that this assumption does not hold if $\tau_{i}$s are random observations over the status of the system, and $X_{i}$s and $Y_{i}$s at $\tau_{i}$ would then strictly depend on $\Delta_{i}$s.

Consequently,

(15) $$\gamma \left(y,z,\theta \right)=Ez^{Y}E\left[y^{X}e^{-\theta \Delta }\right]=a\left(z\right)g\left(y,\theta \right)=\left(az^{N}+bz+c\right)\left[\alpha \left(\theta \right)y+\beta \left(\theta \right)\right].$$

**Example** **1.** *To illustrate our settings in a practical formation of the joint distribution of* Δ *and X, suppose the* Δ*-marginal distribution of $\left(X,\Delta \right)$ is exponential with parameter γ, that is, $\Delta \in \left[\mathrm{Exp}\left(\gamma \right)\right]$. Then, from (12),*

(16) $$\alpha \left(\theta \right)=Ee^{-\theta \Delta }1_{\left\{\Delta <\delta \right\}}=\gamma \int_{x=0}^{\delta }e^{-(\theta +\gamma )x}dx=\frac{\gamma }{\gamma +\theta }\left[1-e^{-\left(\gamma +\theta \right)\delta }\right]$$

*and*

(17) $$\beta \left(\theta \right)=E1_{\left\{\Delta \geq \delta \right\}}e^{-\theta \Delta }=\frac{\gamma }{\gamma +\theta }-\frac{\gamma }{\gamma +\theta }\left[1-e^{-\left(\gamma +\theta \right)\delta }\right]=\frac{\gamma }{\gamma +\theta }e^{-\left(\gamma +\theta \right)\delta }.$$

*Then,*

(18) $$\alpha =\alpha \left(0\right)=P\left\{\Delta <\delta \right\}=1-e^{-\gamma \delta }\;\mathrm{and}\;\beta =\beta \left(0\right)=e^{-\gamma \delta }.$$

*Hence, from (11),*

(19) $$\begin{array}{cc} g\left(y,\theta \right) & =Ey^{X}e^{-\theta \Delta }=Ey^{1_{\left\{\Delta <\delta \right\}}}e^{-\theta \Delta }=\alpha \left(\theta \right)y+\beta \left(\theta \right)\\ \; & =\frac{\gamma }{\gamma +\theta }\left[y\left(1-e^{-\left(\gamma +\theta \right)\delta }\right)+e^{-\left(\gamma +\theta \right)\delta }\right] \end{array}$$

*verifying that the corresponding marginal transforms are*

$$g\left(1,\theta \right)=Ee^{-\theta \Delta }=\frac{\gamma }{\gamma +\theta }\;\;\mathrm{and}\;\;g\left(y,0\right)=Ey^{X}=\alpha y+\beta =\left(1-e^{-\gamma \delta }\right)y+e^{-\gamma \delta }$$

*as it should be. Furthermore, from (19),*

$$\frac{d}{dy}g\left(y,\theta \right){|}_{y=1}=\frac{\gamma }{\gamma +\theta }\left(1-e^{-\left(\gamma +\theta \right)\delta }\right)=\frac{\gamma }{\gamma +\theta }\left(1-e^{-\gamma \delta }e^{-\theta \delta }\right),$$

*we obtain the subcovariance*

$$R\left(\Delta ,X\right)=E\left[\Delta \cdot X\right]=\left(-1\right)\frac{\partial }{\partial \theta }g\left(1,\theta \right){|}_{\theta =0}=\frac{1}{\gamma }\left(1-e^{-\gamma \delta }\right)+\delta e^{-\gamma \delta }.$$

*Therefore, from (19),*

(20) $$\begin{array}{c} Cov\left(\Delta ,X\right)=E\left[\Delta \cdot X\right]-E\Delta \cdot EX\\ =\frac{1}{\gamma }\left(1-e^{-\gamma \delta }\right)+\delta e^{-\gamma \delta }-\frac{1}{\gamma }\left(1-e^{-\gamma \delta }\right)=\delta e^{-\gamma \delta }. \end{array}$$

*The same construction as above can be applied to any absolutely continuous, a.s. positive r.v. with a density f. Of course it would be preferable, although not mandatory, that*

$$\alpha \left(\theta \right)=Ee^{-\theta \Delta }1_{\left\{\Delta <\delta \right\}}=\int_{x=0}^{\delta }e^{-\theta x}f\left(x\right)dx$$

*yields a closed-form expression. For example, a gamma r.v. with parameters $\left(\alpha =r,\beta =\gamma \right),$ where r∈N, will do the job. Its density is $f\left(x\right)=\gamma e^{-\gamma x}\frac{{\left(\gamma x\right)}^{r-1}}{(r-1)!}$, implying that*

$$\alpha \left(\theta \right)=Ee^{-\theta \Delta }1_{\left\{\Delta <\delta \right\}}=\frac{\gamma^{r}}{\left(r-1\right)!}\int_{x=0}^{\delta }e^{-(\theta +\gamma )x}x^{r-1}dx.$$

*The integral can be easily computed because r∈N. For example, for r=3 (without loss of generality),*

$$\begin{array}{c} \alpha \left(\theta \right)=\frac{\gamma^{3}}{2}\int_{x=0}^{\delta }e^{-(\theta +\gamma )x}x^{2}dx=\left[1-e^{-\left(\theta +\gamma \right)\delta }\right]{\left(\frac{\gamma }{\gamma +\theta }\right)}^{3}-\delta \gamma {\left(\frac{\gamma }{\gamma +\theta }\right)}^{2}-\frac{1}{2}{\left(\delta \gamma \right)}^{2}\frac{\gamma }{\gamma +\theta }\\ \beta \left(\theta \right)={\left(\frac{\gamma }{\gamma +\theta }\right)}^{3}-\alpha \left(\theta \right)=e^{-\left(\theta +\gamma \right)\delta }{\left(\frac{\gamma }{\gamma +\theta }\right)}^{3}+\delta \gamma {\left(\frac{\gamma }{\gamma +\theta }\right)}^{2}-\frac{1}{2}{\left(\delta \gamma \right)}^{2}\frac{\gamma }{\gamma +\theta }\\ \alpha \left(0\right)=1-e^{-\gamma \delta }-\delta \gamma e^{-\gamma \delta }-\frac{1}{2}e^{-\gamma \delta }{\left(\delta \gamma \right)}^{2}\\ \beta \left(0\right)=e^{-\gamma \delta }+\delta \gamma e^{-\gamma \delta }+\frac{1}{2}e^{-\gamma \delta }{\left(\delta \gamma \right)}^{2}. \end{array}$$

Now, with the sequence

(21) $$A_{n}=\sum_{k=1}^{n}X_{k},\;n=1,2,\ldots ,$$

of partial sums, we define the ruin index on *M* occasions of pairs of shocks hitting the system within a small time interval:

(22) $$\mu =min\left\{m:A_{m}=M\right\}.$$

Finally, the cumulative ruin index

(23) ρ=μ∧ν

forms the time-to-failure of the system $\tau_{\rho }$ or equivalently, the lifetime of the system. Consequently, $\tau_{\rho }$ is the earliest time of an arriving shock when a total of *M* δ-shocks land in the system or the number of critical shocks reaches *N* or when the arriving shock at $\tau_{\rho }$ is of magnitude $W_{\rho }>H_{2},$ whichever of the three named events comes first.

**Figure 1:**
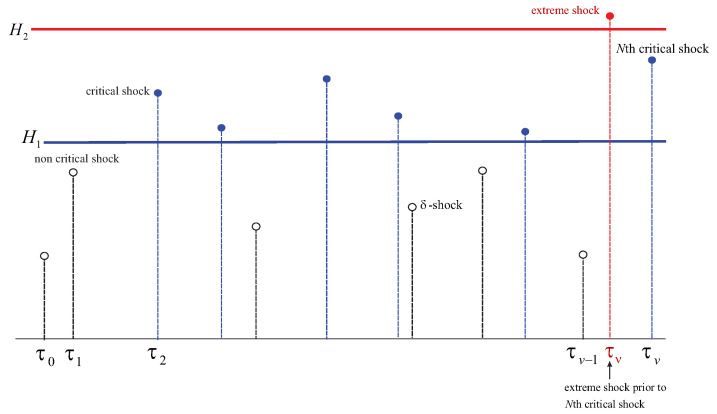
System’s failure due to extreme or *N*th critical shock. Here, ρ=ν.

Figure 1 below depicts system’s failure caused by one of the two conditions: an occurring extreme shock or the number of critical shocks reaching *N*. Here, ρ=ν. This is because μ<M.

**Figure 2:**
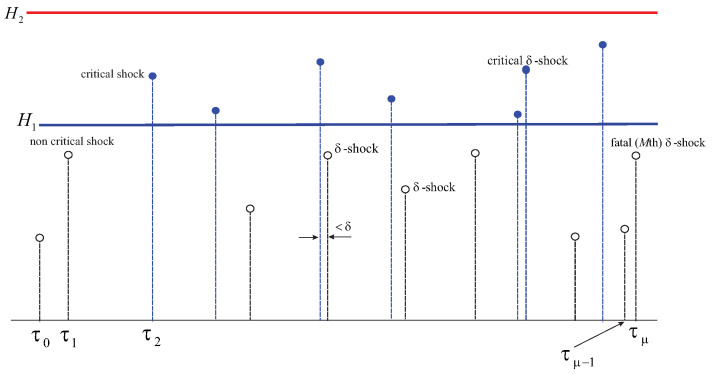
Failure due to *M*th δ-shock that occurs prior to an extreme or *N*th critical shock.

In Figure 2, the cause of the failure is *M* δ-shocks at $\tau_{\mu }$ occurring earlier than $\tau_{\nu }$ (that is, when the total of critical shocks reaches *N* or if an extreme shock strikes). Thus, ρ=μ.

## 3. Background on Discrete Operational Calculus and Its Use for Bivariate Marked Point Processes

In this section, we continue to formalize the above model and lay a foundation for its analysis which goes back to Dshalalow’s article [35] (preceded even by their earlier work), further embellished in Dshalalow and White [36] as well as in this paper. Let $\left(\Omega ,F,\left(F_{t}\right),P\right)$ be a filtered probability space. Given the sequence $\left(X_{k},Y_{k}\right)$ of shock identifiers at respective times $(\tau_{k}),$ we define the random measure with bivariate marks

$$\left(A,B,\tau \right)=\sum_{k=1}^{\infty }\left(X_{k},Y_{k}\right)\varepsilon_{\tau_{k}}\left(\varepsilon_{a}\;\;\mathrm{is}\;\mathrm{the}\;\mathrm{Dirac}\;\mathrm{point}\;\mathrm{mass}\;\mathrm{at}\;a\;\mathrm{point}\;a\right),$$

adapted to filtration $\left(F_{t}\right),$ representing the stream $\left(\tau_{k}\right)$ of shocks and their respective damages to the system. For example, given a set $S\subseteq R_{+},$ the r.v. $\left(A,B,\tau \right)\left(S\right)$ gives the total amount of casualties to the system on time set *S* that can be deduced from $\left(X_{k},Y_{k}\right)$’s involving those *k*s for which $\tau_{k}$s∈S. Most pertinently, if S=[0,t], we can (at least theoretically) conclude whether or not the system remains operational by time *t*. Thus, $\left\{\tau_{k}\right\}$ is a sequence of stopping times relative to $\left(F_{t}\right)$ and so is $\tau_{\rho }$.

Our analysis will focus on the time-to-failure $\tau_{\rho }$, that is, when the system fails due to a fatality through a single extreme shock or due to other damages by all critical or δ-shocks consolidated by $\tau_{\rho }$. Thus, we target the principal portion of $\left(A,B,\tau \right)$ reduced to

$$\left(A_{\rho },B_{\rho },\tau_{\rho }\right)=\sum_{k=1}^{\rho }\left(X_{k},Y_{k}\right)\varepsilon_{\tau_{k}},$$

that is, in the time interval $\left[0,\tau_{\rho }\right]$.

The main purpose is to find the joint distribution of $\tau_{\rho }$ and the cumulative damages to the system at $\tau_{\rho }$ to assess the situation, for example, to see if the overall damage can be fixed and maintained or the system needs replacement. Perhaps it could be reasonable to calibrate the associated thresholds $H_{1},H_{2},$ and δ. An associated control or optimization is more readily feasible if the outcome yields closed-form functionals.

We therefore introduce the functional

Φρs,y,z,θ,u,v,ϑ;M,N=EsρyAρzBρuAρ−1vBρ−1e−θτρ−ϑτρ−1,s≤1,y≤1,z≤1,u≤1,v≤1,Reθ≥0,Reϑ≥0,

to provide comprehensive information on the system at time $\tau_{\rho },$ including ρ (the total shock count upon failure including critical, harmless, extreme, and δs); $A_{\rho }$$\left(\leq M\right)$—the total number of δ-shocks; and $B_{\rho }$—the number of critical and extreme shocks combined. With $B_{\rho }<N,$ the system’s failure is entirely due to $A_{\rho }=M;$ with $B_{\rho }=N,\;$ the system’s failure is due to *N* critical shocks but no extreme shock; and with $B_{\rho }>N,$ the system fails due to a combination of one extreme and critical shocks. Furthermore, if needed, the above functional also provides the information on all named characteristics at time $\tau_{\rho -1},$ that is, upon an epoch of a nonfatal shock preceding the one at time-to-failure $\tau_{\rho }$ of the system.

Other than $\Phi_{\rho },$ of interest is also

$$\Phi_{\mu >\nu }\left(s,y,z,\theta ,s,u,v,\vartheta ;M,N\right)=Es^{\rho }y^{A_{\rho }}z^{B_{\rho }}u^{A_{\rho -1}}v^{B_{\rho -1}}e^{-\theta \tau_{\rho }-\vartheta \tau_{\rho -1}}1_{\mu >\nu }$$

on the confined space $\left(\Omega ,F\cap \left\{\nu <\mu \right\},P_{\left\{\nu <\mu \right\}}\right)$. It gives the status of the system that fails entirely due to critical shocks alone or a combination of critical shocks and one single extreme shock at $\tau_{\rho },$ but not due to δ-shocks. More on this and other variants is established in Theorem 1.

We recall that the random measure $\left(A,B,\tau \right)=\sum_{k=0}^{\infty }\left(X_{k},Y_{k}\right)\varepsilon_{\tau_{k}}$ is a marked point process. More specifically, $\left(A,B,\tau \right)$ is a marked delayed renewal process with position-dependent marking (although it is not required in the Model 1 setting, where we assume position independence). The latter means that

(*i*) $\left(X_{k},Y_{k},\Delta_{k}=\left(\tau_{k}-\tau_{k-1}\right)\right),k=0,1,2,\ldots ,\;\tau_{-1}=0,$ is a sequence of independent random vectors.
(*ii*) The vectors $\left(X_{k},Y_{k},\Delta_{k}\right)\subseteq \left[(X,Y,\Delta )\right],k=1,2,\ldots ,\;$ (that is, identically distributed).
(*iii*) $\left(X_{0},Y_{0},\Delta_{0}=\tau_{0}\right)\notin \left[(X,Y,\Delta )\right]$ (so far no assumption on the initial condition).
(*iv*) The vectors $\left(X_{k},Y_{k}\right)$ may depend on $\Delta_{k}$ but do not depend on $\Delta_{0},\ldots ,\Delta_{k-1},\;k=1,2,\ldots ;\left(X_{0},Y_{0}\right)$ depends on $\Delta_{0}$ (position dependence).

Throughout the rest of the paper, we use the D-operator and its calculus, introduced earlier in Dshalalow [35]. The D-operator, like the differential operator, is parametric (with integer parameter k∈Z), defined as

$$D_{x}^{k}F\left(x,y\right)=\left\{\begin{array}{cc} \lim_{x\to 0}\frac{1}{k!}\frac{\partial^{k}}{\partial x^{k}}\;\left[\;\frac{1}{1-x}\;F\left(x,y\right)\right], & k\geq 0\\ 0, & k<0 \end{array}\right.$$

if it is applied to a function *F* analytic at zero in variable *x*.

As rendered in calculus, where we rarely use the definition of the derivative, we make use of some properties of the D-operator (see [35,36]):

(*Di*) D is a linear functional.
(*Dii*) $D_{x}^{k}\left(1\left(x\right)\right)=1,\;$where 1(x)=1 for all x∈R.
(*Diii*) Let *g* be an analytic function at zero. Then, it holds true that
  $$D_{x}^{k}\left(x^{j}g\left(x\right)\right)=D_{x}^{k-j}g\left(x\right).$$
(*Div*) In particular, if j=k, we have
  $$D_{x}^{k}\left(x^{k}g\left(x\right)\right)=g\left(0\right).$$

**Theorem** **1.** *Let*

(24) $$\left(A,B,\tau \right)=\sum_{k=0}^{\infty }\left(X_{k},Y_{k}\right)\varepsilon_{\tau_{k}}$$

*be a marked random measure with position-dependent marking representing a delayed marked renewal process terminated at $\tau_{\rho }$ such that the joint transforms of the respective increments of $\left(A,B,\tau \right)$ are*

(25) $$\gamma \left(y,z,\theta \right)=Ey^{X}z^{Y}e^{-\theta \Delta }of\;\left(X_{i},Y_{i},\Delta_{i}\right),i=1,2,\ldots ,$$

(26) $$\gamma_{0}\left(y,z,\theta \right)=Ex^{X_{0}}z^{Y_{0}}e^{-\theta \Delta_{0}},$$

[*since $\left(Y_{0},\Delta_{0}\right)$ has a different distribution from $\left(Y_{i},\Delta_{i}\right)$s*], *with the respective components*

(27) $$\Delta_{1}=\tau_{1},\Delta_{2}=\tau_{2}-\tau_{1},\ldots \in \left[\Delta \right]$$

(28) $$\Delta_{0}=\tau_{0}\notin \left[\Delta \right],$$

(29) $$Y_{i}=1_{E_{2}}+N1_{E_{3}},\;with\;the\;distribution\;a=P\left(E_{3}\right),b=P\left(E_{2}\right),c=P\left(E_{1}\right)\left[\mathrm{see}\;\left(1\right)\right]:$$

(30) $$Y_{0}=B_{0}\;is\;an\;\mathrm{integer}\text{-}\mathrm{valued},\;nonnegative,\;r.v.$$

*Then the functionals $\Phi_{\rho },\Phi_{\mu >\nu },\Phi_{\mu <\nu },\Phi_{\mu \geq \nu },\Phi_{\mu \leq \nu }$ satisfy the following formulas:*

(31) $$\begin{array}{c} \Phi_{\rho }\left(s,y,z,\theta ,s,u,v,\vartheta ;M,N\right)=Es^{\rho }y^{A_{\rho }}z^{B_{\rho }}u^{A_{\rho -1}}v^{B_{\rho -1}}e^{-\theta \tau_{\rho }-\vartheta \tau_{\rho -1}}\\ =D_{w}^{N-1}\circ D_{x}^{M-1}[\gamma_{0}\left(y,z,\theta \right)-\gamma_{0}\left(xy,zw,\theta \right)\\ +\Psi \left[\gamma \left(y,z,\theta \right)-\gamma \left(xy,zw,\theta \right)\right]]\left(M,N\right) \end{array}$$

(32) $$\begin{array}{c} \Phi_{\mu >\nu }\left(s,y,z,\theta ,s,u,v,\vartheta ;M,N\right)=Es^{\rho }y^{A_{\rho }}z^{B_{\rho }}u^{A_{\rho -1}}v^{B_{\rho -1}}e^{-\theta \tau_{\rho }-\vartheta \tau_{\rho -1}}1_{\mu >\nu }\\ =D_{w}^{N-1}\circ D_{x}^{M-1}[\gamma_{0}\left(xy,z,\theta \right)-\gamma_{0}\left(xy,zw,\theta \right)\\ +\Psi \left[\gamma \left(xy,z,\theta \right)-\gamma \left(xy,zw,\theta \right)\right]]\left(M,N\right) \end{array}$$

(33) $$\begin{array}{c} \Phi_{\mu <\nu }\left(s,y,z,\theta ,s,u,v,\vartheta ;M,N\right)=Es^{\rho }y^{A_{\rho }}z^{B_{\rho }}u^{A_{\rho -1}}v^{B_{\rho -1}}e^{-\theta \tau_{\rho }-\vartheta \tau_{\rho -1}}1_{\mu <\nu }\\ =D_{w}^{N-1}\circ D_{x}^{M-1}\left\{\gamma_{0}\left(y,zw,\theta \right)-\gamma_{0}\left(xy,zw,\theta \right)+\Psi \left[\gamma \left(y,zw,\theta \right)-\gamma \left(xy,zw,\theta \right)\right]\right\}\left(M,N\right). \end{array}$$

(34) $$\begin{array}{c} \Phi_{\mu =\nu }\left(s,y,z,\theta ,s,u,v,\vartheta ;M,N\right)=Es^{\rho }y^{A_{\rho }}z^{B_{\rho }}u^{A_{\rho -1}}v^{B_{\rho -1}}e^{-\theta \tau_{\rho }-\vartheta \tau_{\rho -1}}1_{\mu =\nu }\\ =D_{w}^{N-1}\circ D_{x}^{M-1}\{\gamma_{0}\left(y,z,\theta \right)-\gamma_{0}\left(y,zw,\theta \right)+\Psi \left[\gamma \left(y,z,\theta \right)-\gamma \left(y,zw,\theta \right)\right]\\ -\left[\gamma_{0}\left(xy,z,\theta \right)-\gamma_{0}\left(xy,zw,\theta \right)+\Psi \left[\gamma \left(xy,z,\theta \right)-\gamma \left(xy,zw,\theta \right)\right]\right\}\left(M,N\right). \end{array}$$

(35) $$\begin{array}{c} \Phi_{\mu \geq \nu }\left(s,y,z,\theta ,s,u,v,\vartheta ;M,N\right)=Es^{\rho }y^{A_{\rho }}z^{B_{\rho }}u^{A_{\rho -1}}v^{B_{\rho -1}}e^{-\theta \tau_{\rho }-\vartheta \tau_{\rho -1}}1_{\mu \geq \nu }\\ =D_{w}^{N-1}\circ D_{x}^{M-1}\left\{\gamma_{0}\left(y,z,\theta \right)-\gamma_{0}\left(y,zw,\theta \right)+\Psi \left[\gamma \left(y,z,\theta \right)-\gamma \left(y,zw,\theta \right)\right]\right\}\left(T\right) \end{array}$$

(36) $$\begin{array}{c} \Phi_{\mu \leq \nu }\left(s,y,z,\theta ,s,u,v,\vartheta ;M,N\right)=Es^{\rho }y^{A_{\rho }}z^{B_{\rho }}u^{A_{\rho -1}}v^{B_{\rho -1}}e^{-\theta \tau_{\rho }-\vartheta \tau_{\rho -1}}1_{\mu \leq \nu }\\ =D_{w}^{N-1}\circ D_{x}^{M-1}\left\{\gamma_{0}\left(y,z,\theta \right)-\gamma_{0}\left(xy,z,\theta \right)+\Psi \left[\gamma \left(y,z,\theta \right)-\gamma \left(xy,z,\theta \right)\right]\right\}\left(T\right). \end{array}$$

*where*

(37) $$\Psi =\Psi \left(s,y,z,\theta ,u,v,\vartheta ;x,y\right)=\frac{s\gamma_{0}\left(xyu,zvw,\theta +\vartheta \right)}{1-s\gamma \left(xyu,zvw,\theta +\vartheta \right)}.$$

**Proof.**  Introduce the following sequences of random indices:

(38) $$\left\{\mu \left(p\right)=\inf\left\{m:A_{m}>p\right\}:p=0,1,\ldots \right\}$$

(39) $$\left\{\nu \left(q\right)=\inf\left\{n:B_{n}>q\right\}:q=0,1,\ldots \right\}$$

(40) $$\left\{\rho \left(p,q\right)=\mu \left(p\right)\wedge \nu \left(q\right):\left(p,q\right)\in N_{0}^{2}\right\}$$

and

(41) $$H_{12}=H_{12}\left(p,q\right)=\left\{\mu \left(p\right)>\nu \left(q\right):\left(p,q\right)\in N_{0}^{2}\right\}$$

(42) $$H_{21}=H_{21}\left(p,q\right)=\left\{\mu \left(p\right)<\nu \left(q\right):\left(p,q\right)\in N_{0}^{2}\right\}$$

(43) $$H_{11}=H_{11}\left(p,q\right)=\left\{\mu \left(p\right)=\nu \left(q\right):\left(p,q\right)\in N_{0}^{2}\right\}$$

Next, define the associated double sequences of functionals:

(44) $$\begin{array}{c} \Phi_{\rho \left(p,q\right)}\left(s,y,z,\theta ,u,v,\vartheta ;p,q\right)=Es^{\rho \left(p,q\right)}y^{A_{\rho \left(p,q\right)}}z^{B_{\rho \left(p,q\right)}}u^{A_{\rho \left(p,q\right)-1}}v^{B_{\rho \left(p,q\right)-1}}e^{-\theta \tau_{\rho \left(p,q\right)}-\vartheta \tau_{\rho \left(p,q\right)-1}}\\ =Es^{\rho \left(p,q\right)}y^{A_{\rho \left(p,q\right)}}z^{B_{\rho \left(p,q\right)}}u^{A_{\rho \left(p,q\right)-1}}v^{B_{\rho \left(p,q\right)-1}}e^{-\theta \tau_{\rho \left(p,q\right)}-\vartheta \tau_{\rho \left(p,q\right)-1}}\left(1_{H_{12}}+1_{H_{21}}+1_{H_{11}}\right)\\ =\Phi_{\mu \left(p\right)>\nu \left(q\right)}\left(s,y,z,\theta ,u,v,\vartheta ;p,q\right)+\Phi_{\mu \left(p\right)<\nu \left(q\right)}\left(s,y,z,\theta ,u,v,\vartheta ;p,q\right)\\ +\Phi_{\mu \left(p\right)=\nu \left(q\right)}\left(s,y,z,\theta ,u,v,\vartheta ;p,q\right),\left(p,q\right)\in N_{0}^{2}. \end{array}$$

From (44), we first work on $\Phi_{\mu \left(p\right)>\nu \left(q\right)}\left(s,y,z,\theta ,u,v,\vartheta ;p,q\right)$:

(45) $$\begin{array}{c} \Phi_{\mu \left(p\right)>\nu \left(q\right)}\left(s,y,z,\theta ,u,v,\vartheta ;p,q\right)=\\ Es^{\rho \left(p,q\right)}y^{A_{\rho \left(p,q\right)}}z^{B_{\rho \left(p,q\right)}}u^{A_{\rho \left(p,q\right)-1}}v^{B_{\rho \left(p,q\right)-1}}e^{-\theta \tau_{\rho \left(p,q\right)}-\vartheta \tau_{\rho \left(p,q\right)-1}}1_{\mu \left(p\right)>\nu \left(q\right)}\\ =Es^{\nu \left(q\right)}y^{A_{\nu \left(q\right)}}u^{A_{\nu \left(q\right)-1}}z^{B_{\nu \left(q\right)}}v^{B_{\nu \left(q\right)-1}}e^{-\theta \tau_{\nu \left(q\right)}-\vartheta \tau_{\nu \left(q\right)-1}}1_{\mu \left(p\right)>\nu \left(q\right)}\\ =\sum_{j=0}^{\infty }\sum_{k>j}Es^{j}y^{A_{j}}z^{B_{j}}u^{A_{j-1}}v^{B_{j-1}}e^{-\theta \tau_{j}-\vartheta \tau_{j-1}}1_{\left\{\mu \left(p\right)=k,\nu \left(q\right)=j\right\}}. \end{array}$$

To continue, we introduce operator *D* applied to a generic function $\left[N_{0},\bar{B}\left(0,1\right)\subseteq C,f\right],$ where $\bar{B}$$\left(0,1\right)$ is a compact unit ball in C centered at zero,

(46) $$D_{p}\left\{f\left(p\right)\right\}\left(x\right)=\sum_{p=0}^{\infty }x^{p}f\left(p\right)\left(1-x\right),\;x\in B\left(0,1\right).$$

(Here, $\left[{\mathrm{Domain}}_{f},{\mathrm{Codomain}}_{f},f\right]$ is a standard specification of function *f*).

Note that the dummy index *p* attached to *D* is being used for convenience only to indicate which variable (if more than one) it applies to. It can be readily shown that $D^{k}$ of $\left(Di\right)$ is the inverse operator of *D* that can revive *f* if we apply it for every *k*:

(47) $$D_{x}^{k}\left(D_{p}\left\{f\left(p\right)\right\}\left(x\right)\right)=f\left(k\right),k=0,1,\ldots$$

Denote the composition

(48) $$D_{pq}\left(\cdot \right)\left(x,w\right)=D_{q}\circ D_{p}\left(\cdot \right)\left(x,w\right).$$

Now the application of operator $D_{pq}$ to $1_{\left\{\mu \left(p\right)=k,\nu \left(q\right)=j\right\}}$ can be readily proven to yield

(49) $$\begin{array}{c} D_{pq}1_{\left\{\mu \left(p\right)=k,\nu \left(q\right)=j\right\}}\left(x,w\right)=\left(x^{A_{k-1}}-x^{A_{k}}\right)\left(w^{B_{j-1}}-w^{B_{j}}\right)\\ =x^{A_{k-1}}\left(1-x^{X_{k}}\right)w^{B_{j-1}}\left(1-w^{Y_{j}}\right),\;j,k=0,1,\ldots ,\;\mathrm{where}\;A_{-1}=B_{-1}=0. \end{array}$$

Using Fubini’s theorem and noticing that $D_{pq}$ is a linear operator, we obtain from (44)–(49),

$$\begin{array}{c} \Phi_{\mu >\nu }^{*}\left(s,y,z,\theta ,u,v,\vartheta ;x,w\right)=D_{pq}\Phi_{\mu \left(p\right)>\nu \left(q\right)}\left(s,y,z,\theta ,u,v,\vartheta ;p,q\right)\left(x,w\right)\\ =\sum_{j=0}^{\infty }s^{j}\;\sum_{k>j}E\left[y^{A_{j}}z^{B_{j}}u^{A_{j-1}}v^{B_{j-1}}e^{-\theta \tau_{j}-\vartheta \tau_{j-1}}D_{pq}1_{\left\{\mu \left(p\right)=k,\nu \left(q\right)=j\right\}}\left(x,w\right)\right]\\ =\sum_{j=0}^{\infty }s^{j}\;\sum_{k>j}E\left[y^{A_{j}}z^{B_{j}}u^{A_{j-1}}v^{B_{j-1}}e^{-\left(\theta +\vartheta \right)\tau_{j-1}-\theta \Delta_{j}}x^{A_{k-1}}\left(1-x^{X_{k}}\right)w^{B_{j-1}}\left(1-w^{Y_{j}}\right)\right] \end{array}$$

separating independent factors

$$=\sum_{j=0}^{\infty }s^{j}\;\sum_{k>j}E\left[{\left(xyu\right)}^{A_{j-1}}{\left(zvw\right)}^{B_{j-1}}e^{-\left(\theta +\vartheta \right)\tau_{j-1}}{\left(yx\right)}^{X_{j}}z^{Y_{j}}\left(1-w^{Y_{j}}\right)e^{-\theta \Delta_{j}}x^{\sum_{i=j+1}^{k-1}X_{i}}\left(1-x^{X_{k}}\right)\right]$$

and by the independent increments property

(50) $$\begin{array}{c} =\gamma_{0}\left(xy,z,\theta \right)-\gamma_{0}\left(xy,zw,\theta \right)+\gamma_{0}\left(xyu,zvw,\theta +\vartheta \right)\left[\gamma \left(xy,z,\theta \right)-\gamma \left(xy,zw,\theta \right)\right]\\ \times \;s\sum_{j=1}^{\infty }s^{j-1}\gamma^{j-1}\left(xyu,zvw,\theta +\vartheta \right)\\ =\gamma_{0}\left(xy,z,\theta \right)-\gamma_{0}\left(xy,zw,\theta \right)+\frac{s\gamma_{0}\left(xyu,zvw,\theta +\vartheta \right)}{1-s\gamma \left(xyu,zvw,\theta +\vartheta \right)}\left[\gamma \left(xy,z,\theta \right)-\gamma \left(xy,zw,\theta \right)\right]. \end{array}$$

The convergence of the series is due to $∥\gamma \left(xyu,zvw,\theta +\vartheta \right)∥<1$ as established in [36].

Finally, we arrive at Formula (32), proving that

$$\Phi_{\mu >\nu }\left(s,y,z,\theta ,u,v,\vartheta ;M,N\right)=Es^{\rho }y^{A_{\rho }}z^{B_{\rho }}u^{A_{\rho -1}}v^{B_{\rho -1}}e^{-\theta \tau_{\rho }-\vartheta \tau_{\rho -1}}1_{\mu >\nu }$$

$$=D_{w}^{N-1}\circ D_{x}^{M-1}\left[\gamma_{0}\left(xy,z,\theta \right)-\gamma_{0}\left(xy,zw,\theta \right)+\frac{s\gamma_{0}\left(xyu,zvw,\theta +\vartheta \right)}{1-\gamma \left(xyu,zvw,\theta +\vartheta \right)}\left[\gamma \left(xy,z,\theta \right)-\gamma \left(xy,zw,\theta \right)\right]\right]\left(M,N\right).$$

$\Phi_{\mu <\nu }\left(s,y,z,\theta ,u,v,\vartheta ;M,N\right)$ of (33) can be obtained from (32) and (50) by interchanging the roles of μ and ν. Thus, analogous to (50),

(51) $$\begin{array}{c} \Phi_{\mu <\nu }^{*}\left(s,y,z,\theta ,u,v,\vartheta ;x,w\right)=D_{pq}\Phi_{\mu \left(p\right)<\nu \left(q\right)}\left(s,y,z,\theta ,u,v,\vartheta ;p,q\right)\left(x,w\right)\\ =\gamma_{0}\left(y,zw,\theta \right)-\gamma_{0}\left(xy,zw,\theta \right)+\Psi \left[\gamma \left(y,zw,\theta \right)-\gamma \left(xy,zw,\theta \right)\right] \end{array}$$

yielding Formula (33). Now, to obtain (34), we use a similar routine.

Lastly, Formula (31) for $\Phi_{\rho }\left(s,y,z,\theta ,u,v,\vartheta ;M,N\right)=Es^{\rho }y^{A_{\rho }}z^{B_{\rho }}u^{A_{\rho -1}}v^{B_{\rho -1}}e^{-\theta \tau_{\rho }-\vartheta \tau_{\rho -1}}$ follows from summing up expressions (32)–(34) as per (44) and a straightforward algebra. Formulas (35) and (36) are also subject to the summation of the pairs of (32), (34) and (33), (34), respectively.   □

## 4. Reduced Functional

In this section, we discuss special cases that not only are in agreement with popular settings in the reliability literature but also are reducible to very tame formulas in support of our claim of closed-form expressions. First, we drop $A_{\rho -1},B_{\rho -1},$ and $\tau_{\rho -1}$ from $\Phi_{\rho }$ (the reference parameters at the time of a shock prior to system’s failure at $\tau_{\rho }$), even though they might be useful in some applications or even as stand-alone characteristics. Furthermore, we assume M=1, rather than being arbitrary. Under this constraint, the system reduces to the most common variant of δ-shock models. One possible shortcoming of this assumption is due to a single instance of any two shocks hitting one after another within a very short time interval and standing on par with seemingly more serious assaults by a single extreme shock or *N* critical shocks. A practical argument for employing this policy is that the system does not have to be destroyed due to a δ-shock (especially if a δ-shock is formed by two consecutive harmless shocks) but may be paused and evaluated for needed maintenance. Granted, a pair of two consecutive harmless shocks can be harmless, but this is hard to know, let alone that in various real-world systems the true magnitudes of shocks is impossible to even approximate; however, often two shocks hitting one after another within a short time interval can raise flags. Secondly, it is possible that at least one of the two shocks in δ-form can be harmful. Thirdly, we address this issue when constructing Model 2 in Section 7, Section 8, Section 9 and Section 10.

### Main Formula

From (31),

$$\begin{array}{c} \Phi_{\rho }\left(s,y,z,\theta ,u,v,\vartheta ;M,N\right)=Es^{\rho }y^{A_{\rho }}z^{B_{\rho }}u^{A_{\rho -1}}v^{B_{\rho -1}}e^{-\theta \tau_{\rho }-\vartheta \tau_{\rho -1}}\\ =D_{w}^{N-1}\circ D_{x}^{M-1}[\gamma_{0}\left(y,z,\theta \right)-\gamma_{0}\left(xy,zw,\theta \right)\\ +\frac{s\gamma_{0}\left(xyu,zvw,\theta +\vartheta \right)}{1-s\gamma \left(xyu,zvw,\theta +\vartheta \right)}\left[\gamma \left(y,z,\theta \right)-\gamma \left(xy,zw,\theta \right)\right]]\left(M,N\right) \end{array}$$

we drop $A_{\rho -1},B_{\rho -1},$ and $\tau_{\rho -1}$, reducing (31) to the formula for the joint distribution of the lifetime $\tau_{\rho };$ the cumulative damages to the system $A_{\rho },B_{\rho }$ from critical, extreme, or δ-shocks (whichever of the three occurs first) at the failure time; and the total shock count ρ, namely

(52) $$\begin{array}{c} \Phi_{\rho }\left(s,y,z,\theta ,1,1,0;M,N\right)=Es^{\rho }y^{A_{\rho }}z^{B_{\rho }}e^{-\theta \tau_{\rho }}\\ =D_{w}^{N-1}\circ D_{x}^{M-1}[\gamma_{0}\left(y,z,\theta \right)-\gamma_{0}\left(xy,zw,\theta \right)\\ +\frac{s\gamma_{0}\left(xy,zw,\theta \right)}{1-s\gamma \left(xy,zw,\theta \right)}\left[\gamma \left(y,z,\theta \right)-\gamma \left(xy,zw,\theta \right)\right]]\left(M,N\right). \end{array}$$

**Remark** **3.** *Functionals $\Phi_{\rho }$ in Formulas (31) and (52) include a prehistory of the system (that is, prior to the current process of shocks lashing at the system from $\tau_{0}$ until $\tau_{\rho };$ plainly, prior to $\tau_{0}$). It may pertain to a history of prior damages to the system until $\tau_{0}$ that were not reset or repaired and thus had to be integrated as its initial condition. In particular, it can carry out crossings of lower threshold values $M_{0}$ and $N_{0}$.*

*The historical information on the system is included in the initial distribution*

$$\gamma_{0}\left(y,z,\theta \right)=Ey^{X_{0}}z^{Y_{0}}e^{-\theta \tau_{0}}$$

*as the joint transform of $X_{0}$–the δ-shocks count; $Y_{0}$–the number of critical shocks; and the duration $\tau_{0}$ of the process observed from the inception. If any of the specified conditions of system’s failure at $\tau_{0}$ are already met, it will be instantly detected by one of the D-operators pertaining to property $\left(Div\right),$ with no further development past $\tau_{0},$ because the system would be inoperational.*

*Yet, to tame the underlying formulas in Theorem 1, we often set $\tau_{0}=0$ and $X_{0}$ and $Y_{0}$ as constants or zeros serving as sufficiently reasonable initial conditions for the system. As mentioned, however, more comprehensive data can include a full cycle of prior assaults and its outcome that can conveniently be integrated by merging utilizing the flexibility of Formulas (31) and (52). This option in its most general form is always available, but it would extend our current work beyond its length and we choose to postpone it.*

*For now, we reduce the historical process to $X_{0}=Y_{0}=\tau_{0}=0$, implying that $\gamma_{0}=1$. That being said, with $X_{0}=0,$ we have no prior δ-shocks but one harmless shock at $\tau_{0}$, as per our discussion in Remark 2. We recall that all Ys have distribution $c=P\left\{Y=0\right\},b=P\left\{Y=1\right\},$ and $a=P\left\{Y=N\right\}$. We generally assume that a,b,c are positive. However, the latter applies only to $Y_{1},Y_{2},\ldots ,$ and not to $Y_{0}$ which has a=b=0 to enable a δ-shock at $\tau_{1}$ with probability 1 in the event $\left|\left(\tau_{0},\tau_{1}\right)\right|<\delta ,$ as pointed out in Remark 2.*

Consequently, under $\gamma_{0}=1,$ Formula (52) is further reduced to

(53) $$\begin{array}{c} \Phi_{\rho }\left(s,y,z,\theta ,1,1,0;M,N\right)=Es^{\rho }y^{A_{\rho }}z^{B_{\rho }}e^{-\theta \tau_{\rho }}\\ =D_{w}^{N-1}\circ D_{x}^{M-1}\left[\frac{s\gamma \left(y,z,\theta \right)-s\gamma \left(xy,zw,\theta \right)}{1-s\gamma \left(xy,zw,\theta \right)}\right]\left(M,N\right). \end{array}$$

Now, from

$$\frac{s\gamma \left(y,z,\theta \right)-s\gamma \left(xy,zw,\theta \right)}{1-s\gamma \left(xy,zw,\theta \right)}=\frac{1-s\gamma \left(xy,zw,\theta \right)-1+s\gamma \left(y,z,\theta \right)}{1-s\gamma \left(xy,zw,\theta \right)}=1-\frac{1-s\gamma \left(y,z,\theta \right)}{1-s\gamma \left(xy,zw,\theta \right)}$$

and by properties $\left(Dii\right)$ and $\left(Diii\right)$ of operator D, we arrive at an even tamer expression for $\Phi_{\rho }$.

(54) $$\begin{array}{c} \Phi_{\rho }\left(s,y,z,\theta ,1,1,0;M,N\right)=Es^{\rho }y^{A_{\rho }}z^{B_{\rho }}e^{-\theta \tau_{\rho }}\\ \;\;\;\;\;\;=1-\left[1-s\gamma \left(y,z,\theta \right)\right]D_{w}^{N-1}\circ D_{x}^{M-1}\left[\frac{1}{1-s\gamma \left(xy,zw,\theta \right)}\right]\left(M,N\right). \end{array}$$

In a nutshell, the associated special case of (31) in Theorem 1 agrees with the following:

**Corollary** **1.** *Under the conditions of Theorem 1, setting $X_{0}=Y_{0}=\tau_{0}=0$ and dropping $A_{\rho -1},B_{\rho -1},$ and $\tau_{\rho -1},$ the reduced functional $\Phi_{\rho }\left(s,y,z,\theta ,1,1,0;M,N\right)$ satisfies Formula (54).*

As pointed out in the beginning of this section, our next attempt to further reduce $\Phi_{\rho }$ is through setting M=1. We checked out the general case for M≥1 and obtained fully explicit, although bulkier, formulas. Consequently, we decided to postpone and finish it in a stand-alone paper. Now, with M=1, the final variant of (54) turns

$$\begin{array}{cc} \varphi_{\rho }\left(s,y,z,\theta ;N\right) & =\Phi_{\rho }\left(s,y,z,\theta ,1,1,0;1,N\right)\\ \; & =Es^{\rho }y^{A_{\rho }}z^{B_{\rho }}e^{-\theta \tau_{\rho }}\\ \; & =1-\left[1-s\gamma \left(y,z,\theta \right)\right]D_{w}^{N-1}\circ D_{x}^{0}\left[\frac{1}{1-s\gamma \left(xy,zw,\theta \right)}\right] \end{array}$$

using $\left(Dv\right)$

(55) $$=1-\left[1-s\gamma \left(y,z,\theta \right)\right]D_{w}^{N-1}\frac{1}{1-s\gamma \left(0,zw,\theta \right)}.$$

Thus,

(56) $$\begin{array}{c} \varphi_{\rho }\left(s,y,z,\theta ;N\right)=Es^{\rho }y^{A_{\rho }}z^{B_{\rho }}e^{-\theta \tau_{\rho }}\\ \;\;\;\;\;\;=1-\left[1-s\gamma \left(y,z,\theta \right)\right]\Pi \left(s,z,\theta \right) \end{array}$$

where

$$\Pi \left(s,z,\theta \right)=D_{w}^{N-1}\frac{1}{1-s\gamma \left(0,zw,\theta \right)}.$$

From (15), where $\gamma \left(y,z,\theta \right)=\left(az^{N}+bz+c\right)\left[\alpha \left(\theta \right)y+\beta \left(\theta \right)\right]$, we have

$$\frac{1}{1-s\gamma \left(xy,zw,\theta \right)}=\frac{1}{1-s\left(az^{N}w^{N}+bzw+c\right)\left[\alpha \left(\theta \right)xy+\beta \left(\theta \right)\right]}\;\;$$

implying that

(57) $$\frac{1}{1-s\gamma \left(0,zw,\theta \right)}=\frac{1}{1-s\beta \left(\theta \right)\left(az^{N}w^{N}+bzw+c\right)}.$$

After a straightforward algebra and abbreviations,

(58) $$A=A\left(s,z,\theta \right)=\frac{sa\beta \left(\theta \right)z^{N}}{1-sc\beta \left(\theta \right)},\;B=B\left(s,z,\theta \right)=\frac{sb\beta \left(\theta \right)z}{1-sc\beta \left(\theta \right)},\;C=C\left(s,\theta \right)=\frac{1}{1-sc\beta \left(\theta \right)}$$

we arrive at

(59) $$\frac{1}{1-s\gamma \left(0,zw,\theta \right)}=C\frac{1}{1-\left(Aw^{N}+Bw\right)}.$$

To proceed with operator $D_{w}^{N-1}$, we expand $C\frac{1}{1-\left(Aw^{N}+Bw\right)}$ in the functional series

$$\pi =\pi \left(s,zw,\theta \right)=C\sum_{n=0}^{\infty }{\left[Aw^{N}+Bw\right]}^{n}$$

that agrees with $C\frac{1}{1-\left(Aw^{N}+Bw\right)}$ on an open ball $B\left(0,\varepsilon \right)\subseteq C$ of radius ε centered at w=0, where $C\frac{1}{1-\left(Aw^{N}+Bw\right)}$ is analytic. π is a feasible representation of $C\frac{1}{1-\left(Aw^{N}+Bw\right)}$ on $B\left(0,\varepsilon \right),$ because operator D requires exactly that.

To continue, we rewrite π in the form

$$\pi =C\sum_{n=0}^{\infty }{\left[Aw^{N-1}+B\right]}^{n}w^{n}.$$

Then, applying $D_{w}^{N-1}$ toπunder $\left(Div\right)$ gives

(60) $$\Pi \left(s,z,\theta \right)=D_{w}^{N-1}\pi =C\sum_{n=0}^{N-1}D_{w}^{N-1-n}{\left[Aw^{N-1}+B\right]}^{n}.$$

The next step is due to the following:

**Lemma** **1.** *For n=0,1,…R, it holds true that*

$$D_{w}^{R-n}{\left(aw^{R}+b\right)}^{n}=b^{n}.$$

**Proof.**  Firstly,

DwR−nawR+bn=∑k=0nnkakbn−kDwR−nwRk.

Then,

$$D_{w}^{R-n}{\left(aw^{R}+b\right)}^{n}=1=b^{0},\mathrm{if}\;n=0$$

Further, for k≤n,n≥1,

$$D_{w}^{R-n}w^{Rk}=1,\;\mathrm{if}\;k=0\;\mathrm{and}\;D_{w}^{R-n}w^{Rk}=0,\;\mathrm{if}\;k>0$$

Thus, DwR−nawR+bn=∑k=0nnkakbn−k10k=bn.   □

So, from Lemma 1 and Equation (60),

(61) $$\begin{array}{cc} \Pi \left(s,z,\theta \right) & =C\sum_{n=0}^{N-1}B^{n}=C\frac{1-B^{N}}{1-B}=\frac{1}{1-sc\beta \left(\theta \right)}\frac{1-B^{N}}{1-\frac{sb\beta \left(\theta \right)z}{1-sc\beta \left(\theta \right)}}=\frac{1-B^{N}}{1-sc\beta \left(\theta \right)-sb\beta \left(\theta \right)z}\\ \; & =\frac{1}{1-s\beta \left(\theta \right)\left(c+bz\right)}\left[1-{\left(\frac{sb\beta \left(\theta \right)z}{1-sc\beta \left(\theta \right)}\right)}^{N}\right]. \end{array}$$

In particular,

(62) $$\Pi =\Pi \left(1,1,0\right)=\frac{1-B^{N}}{1-\left(1-a\right)\beta }=\frac{1-B^{N}}{\alpha +a\beta }=\frac{1}{\alpha +a\beta }\left[1-{\left(\frac{b\beta }{1-c\beta }\right)}^{N}\right],$$

which will play a key role in the forthcoming sections.

Thus, from (4), (11), (55) and (61),

(63) $$\begin{array}{c} \varphi_{\rho }\left(s,y,z,\theta ;N\right)=Es^{\rho }y^{A_{\rho }}z^{B_{\rho }}e^{-\theta \tau_{\rho }}\\ =1-\left[1-s\left\{\alpha \left(\theta \right)y+\beta \left(\theta \right)\right\}a\left(z\right)\right]\left\{\frac{1}{1-s\beta \left(\theta \right)\left(c+bz\right)}\left[1-{\left(\frac{sb\beta \left(\theta \right)z}{1-sc\beta \left(\theta \right)}\right)}^{N}\right]\right\}, \end{array}$$

herewith arriving at a fully explicit expression.

In conclusion:

**Corollary** **2.** *Under the conditions of Theorem 1, with $X_{0}=Y_{0}=\tau_{0}=0,$ dropping $A_{\rho -1},B_{\rho -1},$ and $\tau_{\rho -1},$ and reducing the number of δ-shocks M to one, the functional $\Phi_{\rho }\left(s,y,z,\theta ,1,1,0;1,N\right)=\varphi_{\rho }\left(s,y,z,\theta ;N\right)$ satisfies Formula (63).*

**Example** **2.** *The functional $\varphi_{\rho }\left(s,y,z,\theta ;N\right)=Es^{\rho }y^{A_{\rho }}z^{B_{\rho }}e^{-\theta \tau_{\rho }}$ in (63) represents a closed-form expression, which is obvious, and it is reducible to a fully explicit formula once $\alpha \left(\theta \right)$ and $\beta \left(\theta \right)$ are specified. We turn to Example 1, with    *

$$\Delta \in \left[\mathrm{Exp}\left(\gamma \right)\right]and\;\alpha \left(\theta \right)=\frac{\gamma }{\gamma +\theta }\left[1-e^{-\left(\gamma +\theta \right)\delta }\right],\;\beta \left(\theta \right)=\frac{\gamma }{\gamma +\theta }e^{-\left(\gamma +\theta \right)\delta }$$

*that can be substituted in (63), while $a\left(z\right)=az^{N}+bz+c$ is all set.*

## 5. Marginal Distributions and Means

From Formula (63) for the joint distribution of the time-to-failure $\tau_{\rho }$ and other characteristics of system’s failure, we obtain marginal transforms starting with $\tau_{\rho }$.

### 5.1. Time-to-Failure

For s=y=z=1, we arrive at

(64) $$Ee^{-\theta \tau_{\rho }}=\varphi_{\rho }\left(1,1,1,\theta ;N\right)=1-\left[1-\gamma \left(\theta \right)\right]\Pi \left(1,1,\theta \right),$$

where

(65) $$\Pi \left(1,1,\theta \right)=\left\{\frac{1}{1-\beta \left(\theta \right)\left(b+c\right)}\left[1-{\left(\frac{b\beta \left(\theta \right)}{1-c\beta \left(\theta \right)}\right)}^{N}\right]\right\}$$

as per (61).

The mean of $\tau_{\rho }$ can be easily derived from (62), (64) and (65):

(66) $$E\tau_{\rho }=\frac{1}{\gamma }\Pi =\frac{1}{\gamma }\frac{1}{\alpha +a\beta }\left[1-{\left(\frac{b\beta }{1-c\beta }\right)}^{N}\right].$$

**Figure 3:**
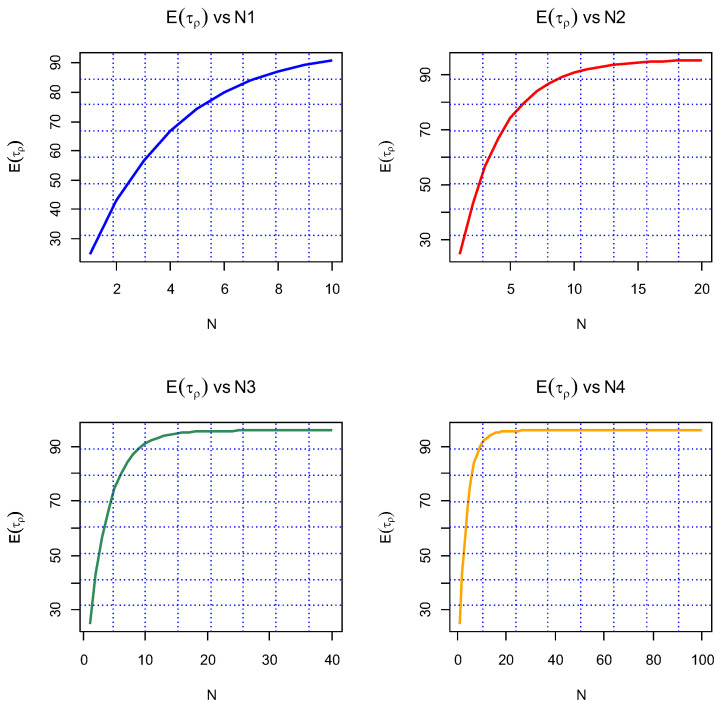
The figure depicts $E\tau_{\rho }$ under fixed a=0.1, b=0.3, c=0.6, γ=0.1, $\delta =0.1,\;\alpha =1-e^{-\gamma \delta },$ and β=1−α and with four different maximum values for *N*: 10,20,40,and 100.

Figure 3 below depicts $E\tau_{\rho }\left(N\right)$ in *N*, ranging from 1 to 100 with four different scales, allowing us to see with what speed $E\tau_{\rho }$ approaches a constant value. It looks like it reaches equilibrium for *N* around 50 under a fixed choice of main parameters.

**Figure 4:**
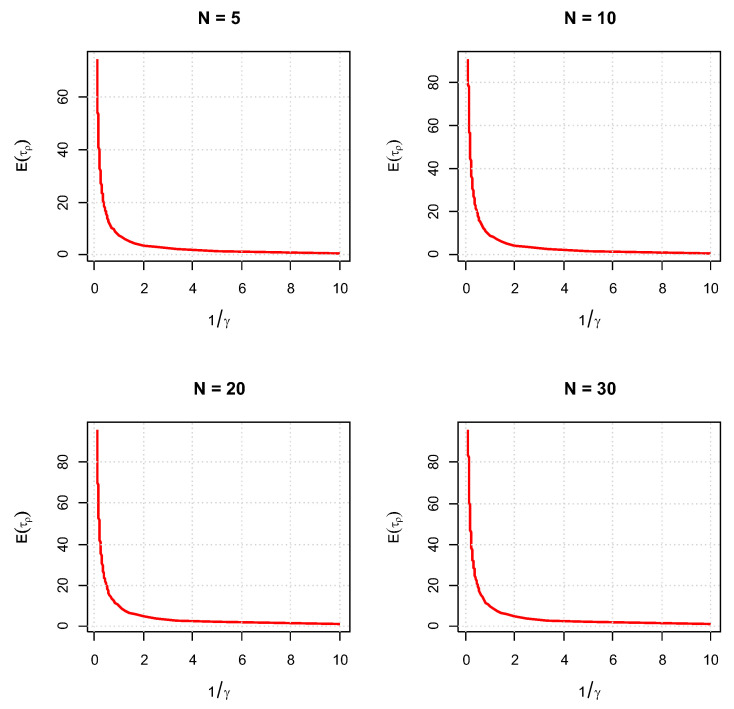
The figure depicts $E\tau_{\rho }$ as a function of $\frac{1}{\gamma }$ running from 1 to 10 under fixed a=0.1, b=0.3, c=0.6, $\delta =0.05,\;\alpha =1-e^{-\gamma \delta },$ β=1−α with four fixed values for *N*: 5,10,20,30.

Figure 4 takes on $E\tau_{\rho }$ as a function of $\frac{1}{\gamma }$ in the interval $\left(0.1,10\right)$ for four different fixed *N*s, 5,10,20,30. Recall that $\frac{1}{\gamma }$ is the mean time between any two consecutive shocks. The rest of the parameters are fixed. We see that $E\tau_{\rho }\left(\frac{1}{\gamma }\right)$ is monotone-decreasing.

### 5.2. Assessment of (66)

We render a quick verification in (66) that ${\left(\frac{b\beta }{1-c\beta }\right)}^{N}<1$ under the assumptions that 0<a and 0<α. Indeed, let ∗ be one of the relations <,≤,=,>,≥. Then, because cβ<1 (or else a=b=α=0),

$$\frac{b\beta }{1-c\beta }*1\Leftrightarrow \left(b+c\right)\beta *1\;\;$$

implying that

$$\left(b+c\right)\beta <\left(a+b+c\right)\beta \leq 1\;\mathrm{or}\;\left(b+c\right)\beta \leq \left(a+b+c\right)\beta <1.$$

It follows that relation ∗ is < and thus

(67) $${\left(\frac{b\beta }{1-c\beta }\right)}^{N}<1.$$

Because of (67), $E\tau_{\rho }$ is monotone-increasing in N, with the largest value $\frac{1}{\gamma }\frac{1}{\alpha +a\beta }$ at N=∞ (see Remark 4 below regarding N=∞) and the smallest at N=1:

(68) $$E\tau_{\rho }\left(1\right)=\frac{1}{\gamma }\frac{1}{\alpha +a\beta }\frac{\alpha +a\beta }{1-c\beta }=\frac{1}{\gamma }\frac{1}{1-c\beta }$$

(due to $1-\frac{b\beta }{1-c\beta }=\frac{\alpha +a\beta }{1-c\beta }$)

(69) $$E\tau_{\rho }\left(\infty \right)=\frac{1}{\gamma }\frac{1}{\alpha +a\beta },$$

respectively. The mean length of the lifetime $\tau_{\rho }$ depends on the mean interarrival time $\frac{1}{\gamma }$ of shocks and on $\alpha =P\left\{\Delta <\delta \right\}$ and β=1−α, but more on α. With α small and β large, α+aβ gets smaller, and thus $\frac{1}{\gamma }\frac{1}{\alpha +a\beta }\left[1-{\left(\frac{b\beta }{1-c\beta }\right)}^{N}\right]$ gets generally larger. This is because of a lesser impact of δ-shocks and the competition running more between extreme and critical shocks, with a lesser chance to be interrupted by a single δ-shock.

With *N* large, as per version (69), $E\tau_{\rho }$ is dominated by α alone, where the competition runs entirely between extreme and δ-shocks. Thus, with α small, γ fixed, $E\tau_{\rho }$ largely depends on a single extreme shock. Of course, $E\tau_{\rho }$ in all cases can be made arbitrarily long by decreasing γ and at the same time making any δ-shock’s occurrence unlikely.

**Remark** **4.** *Because the key result of Theorem 1 is exclusively established for a finite N, one needs to take extreme caution with N→∞. In particular, some interpretations under N=∞ may be even inaccurate or contradictory. The meaning of N=∞ in the context of the D-operator at the center of Theorem 1 is reminiscent of improper integrals, which circumvent a rigorous Riemann–Darboux construction on compact intervals and sometimes disagree with direct and Lebesgue integrals yet are often used. For that reason, it would be safer to reason with an asymptotic behavior of respective quantities involved under N very large rather than N=∞.*

### 5.3. δ-Shocks Count

From (63), with s=z=1,θ=0,

(70) $$\begin{array}{c} \varphi_{\rho }\left(1,y,1,0;N\right)=Ey^{A_{\rho }}\\ \;\;\;\;\;\;=1-\left[1-\left\{\alpha y+\beta \right\}\right]\Pi =1-\alpha \left(1-y\right)\Pi \end{array}$$

whereas per (62),

(71) $$\Pi =\left\{\frac{1}{\alpha +a\beta }\left[1-{\left(\frac{b\beta }{1-c\beta }\right)}^{N}\right]\right\},$$

we have the PGF of the δ-shocks count prior to system’s failure. (70) and (71) can be rewritten as

(72) $$Ey^{A_{\rho }}=1-\alpha \Pi +\alpha \Pi y$$

implying that $A_{\rho }$ is Bernoulli with parameter αΠ, which is also the mean of $A_{\rho }$. Obviously, the mean of $A_{\rho }$ is strictly less than 1.

In a nutshell,

(73) $$EA_{\rho }=\alpha \Pi .$$

**Figure 5:**
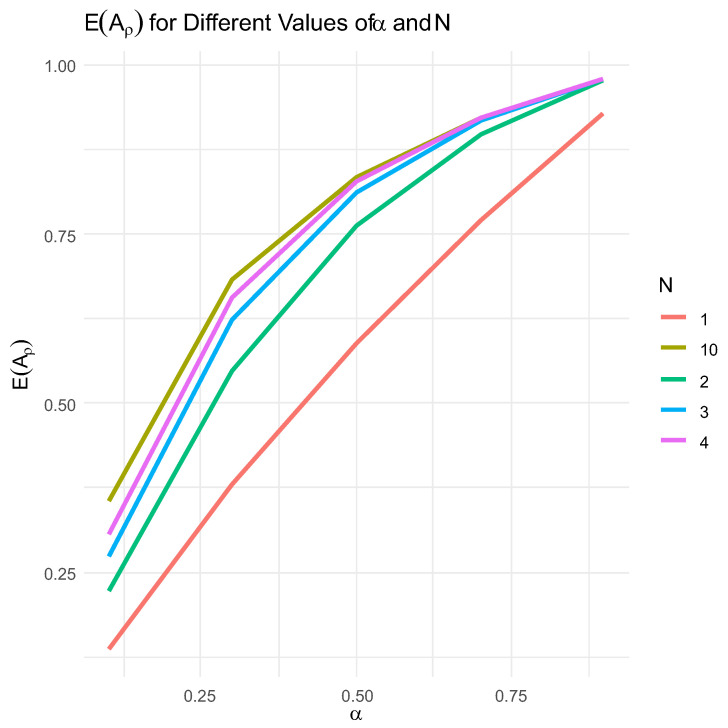
For a=0.2, b=0.5, c=0.3, β=1−α, with 5 different values for *N*, where $EA_{\rho }$ varies in α.

Figure 5 presents five plots of $EA_{\rho }\left(\alpha \right)$, comparing them under five different fixed *N* values. Recall that $\alpha =P\left\{\Delta <\delta \right\}=1-e^{-\gamma \delta }$ when $\Delta \in \left[\mathrm{Exp}\left(\gamma \right)\right]$. To plot the five graphs, we did not specify γ and δ. However, we can keep γ fixed like $\frac{1}{\gamma }=10$ and vary δ in accord with α. Obviously, $\delta =-\frac{1}{\gamma }$ln$\left(1-\alpha \right)$ becomes monotone increasing in α with γ fixed, and so does $EA_{\rho }\left(\alpha \right)$. Consequently, it becomes increasingly more likely to ruin the system with a δ-shock against critical and extreme shocks and we see it in the plots below that $EA_{\rho }$ approaches 1 under *N*s ranging from 1 to 10.

In Figure 5, we draw $EA_{\rho }$ for variable values of α and five variants of N=1, 2, 3, 4, 10 but for fixed a, b, c, where a=0.2, b=0.5, c=0.3, β=1−α.

### 5.4. Critical/Extreme Shocks Damage

From (63), with s=y=1,θ=0,

(74) $$\varphi_{\rho }\left(1,1,z,0;N\right)=Ez^{B_{\rho }}=1-\left[1-a\left(z\right)\right]\Pi \left(1,z,0\right),$$

where

(75) $$\Pi \left(1,z,0\right)=\frac{1}{1-\beta \left(c+bz\right)}\left[1-{\left(\frac{b\beta z}{1-c\beta }\right)}^{N}\right]$$

as per (61). The mean of $B_{\rho }$ can be easily derived from (62), (74) and (75):

(76) $$EB_{\rho }=\left(aN+b\right)\Pi =\frac{aN+b}{\alpha +a\beta }\left[1-{\left(\frac{b\beta }{1-\beta c}\right)}^{N}\right].$$

### 5.5. Total Shock Count

From (63), with y=z=1,θ=0,

(77) $$\varphi_{\rho }\left(s,1,1,0;N\right)=Es^{\rho }=1-\left[1-s\right]\Pi \left(s,1,0\right),$$

where

(78) $$\Pi \left(s,1,0\right)=\frac{1}{1-s\beta \left(c+b\right)}\left[1-{\left(\frac{sb\beta }{1-sc\beta }\right)}^{N}\right]$$

as per (61). The mean of ρ can then be easily derived from (62), (77) and (78) as

(79) $$E\rho =\Pi =\frac{1}{\alpha +a\beta }\left[1-{\left(\frac{b\beta }{1-\beta c}\right)}^{N}\right].$$

**Figure 6:**
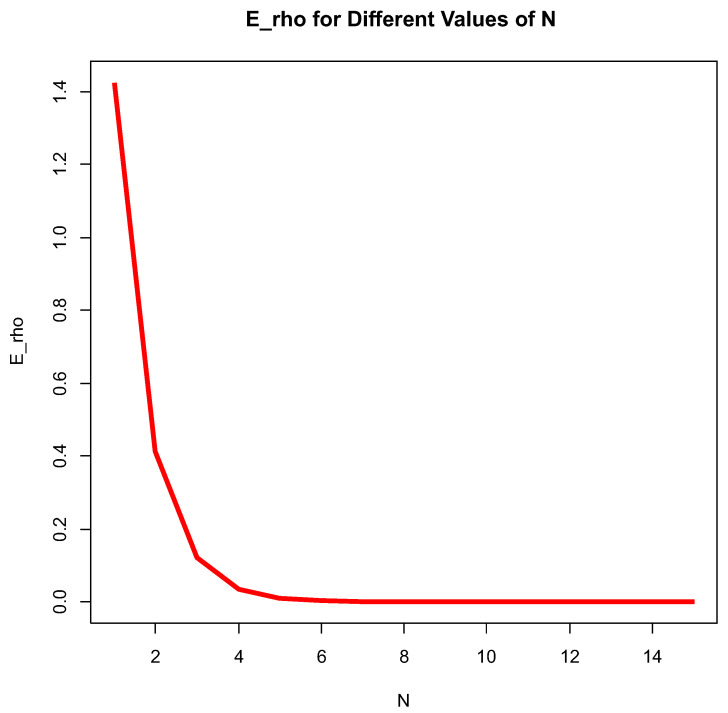
$\gamma =0.1,\;\delta =0.05,\;\alpha =1-e^{-\gamma \delta },\;\beta =1-\alpha ,\;a=0.2,\;b=0.5,\;c=0.3$.

**Figure 7:**
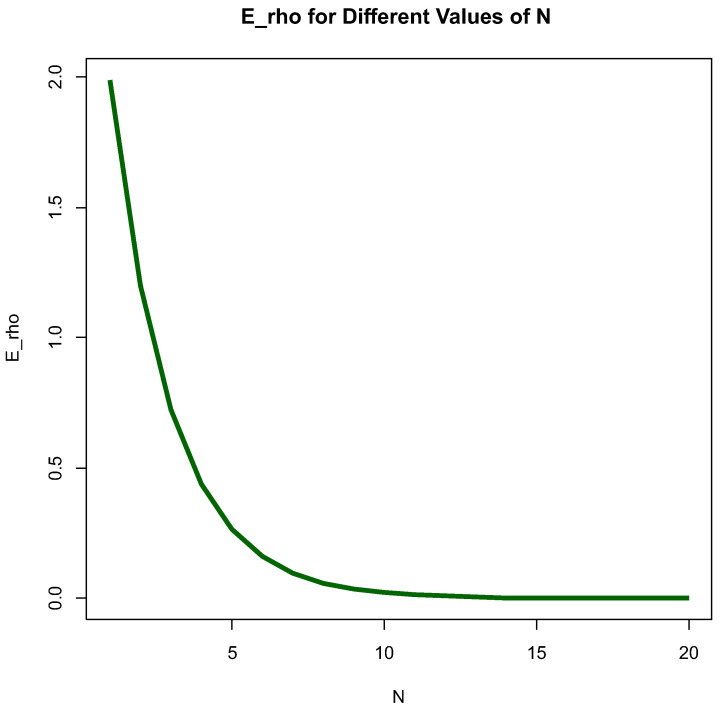
$\gamma =0.1,\;\delta =0.05,\;\alpha =1-e^{-\gamma \delta },\;\beta =1-\alpha ,\;a=0.3,\;b=0.2,\;c=0.5$.

Figure 6 and Figure 7 depict Eρ as a function of *N* with different fixed a, b, c and scales of *N*.

### 5.6. Monte Carlo Simulation of the Process

We next render Monte Carlo simulations of the full stochastic process under some specified special cases and compare empirical means derived above as a demonstration of the results matching empirical findings. In each case below, we assume the times between shocks is are exponential (γ), and we make numerical assumptions about the parameters, including the parameter of time between shocks γ, the time δ, the probabilities of each failure type (*a*, *b*, and *c*), the δ-shock threshold M=1, and the critical shock threshold *N*.

For the first set of experiments, we set γ=δ=1, M=1, and N=2. Figure 3, Figure 4, Figure 5 and Figure 6 below show a comparison of predicted and estimated means of the number of the failure times $\tau_{\rho }$, shocks ρ, δ-shocks $A_{\rho }$ upon failure, and (N×Extreme+Critical) shocks $B_{\rho }$, respectively.

Predicted values come from numerical implementations of (66), (73), (76) and (79) (refer to Appendix A for the Python code). Means are estimated as sample means of each value computed at end-of-life for a sample of 1,000,000 simulated paths (refer to Appendix B for Python implementations for simulations). This experiment is replicated for each (a,b)∈{0.1,0.2,…,0.9}×{0,0.1,…,0.8} with c=1−a−b.

We display three heat maps: predicted mean, sample mean, and absolute difference for each set of probabilities. In every parameter set tested, the error between the true predicted mean and sample mean is less than 0.002, providing a good validation of the predictions derived above.

**Figure 8:**
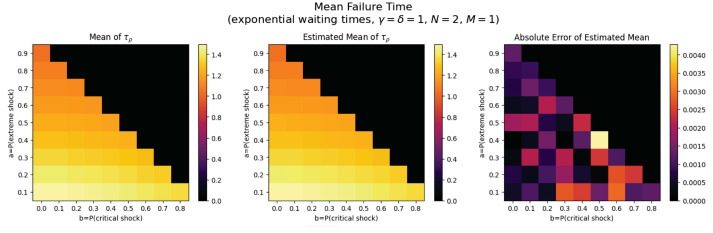
Predicted/estimated mean and absolute error for the failure time $\tau_{\rho }$.

**Figure 9:**
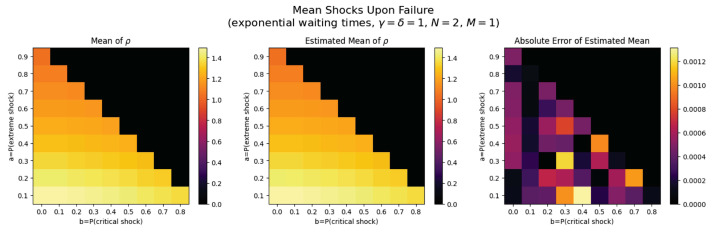
Predicted/estimated mean and absolute error for the shocks ρ.

Note the predicted means of the failure time $\tau_{\rho }$ in Figure 8 and shocks ρ in Figure 9 are identical since EΔ=1 in this case.

**Figure 10:**
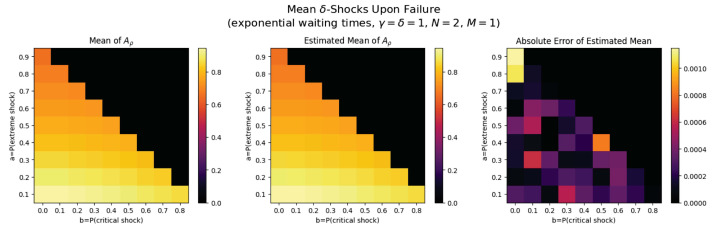
Predicted/estimated mean and absolute error for the δ-shocks $A_{\rho }$.

We notice $\tau_{\rho }$, ρ, and $A_{\rho }$ have broadly the same pattern: an increase to the extreme shock probability *a* results in smaller means. This makes sense because a high *a* indicates a high probability that a single shock knocks down the system, so fewer total shocks are likely to occur over less time with fewer opportunities for δ-shocks. More subtly, increasing *b* has a negative impact on the means for constant *a* because it increases the chance of critical shock failures in fewer total shocks, reducing all three means (See Figure 10).

**Figure 11:**
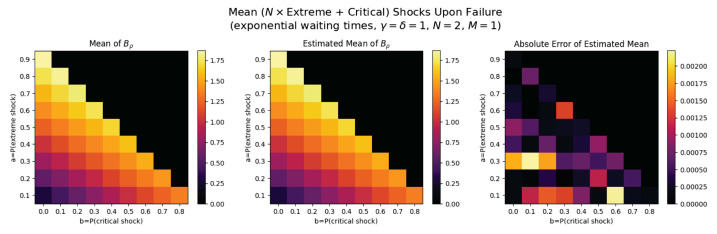
Predicted/estimated mean and absolute error for the (N×Extreme+Critical) shocks $B_{\rho }$.

The trend here is drastically different: $B_{\rho }$ is positively related to both extreme shock probability *a* and critical shock probabilities *b*. This makes sense: if *a* or *b* increase, each shock is more likely to be extreme or critical, each of which add to $B_{\rho }$. Further, when these probabilities are low, δ-shock failures become more likely, in which case $B_{\rho }$ tends to be smaller (See Figure 11).

In addition, we perform some simulations where δ=1,(a, b, c)=(0.5, 0.4, 0.1), and M=1. Furthermore, we vary the waiting time parameter γand critical/extreme shock threshold *N* as

(γ, N)∈{0.1, 0.2, …, 2}×{1, 3, 5, 10}.

**Figure 12:**
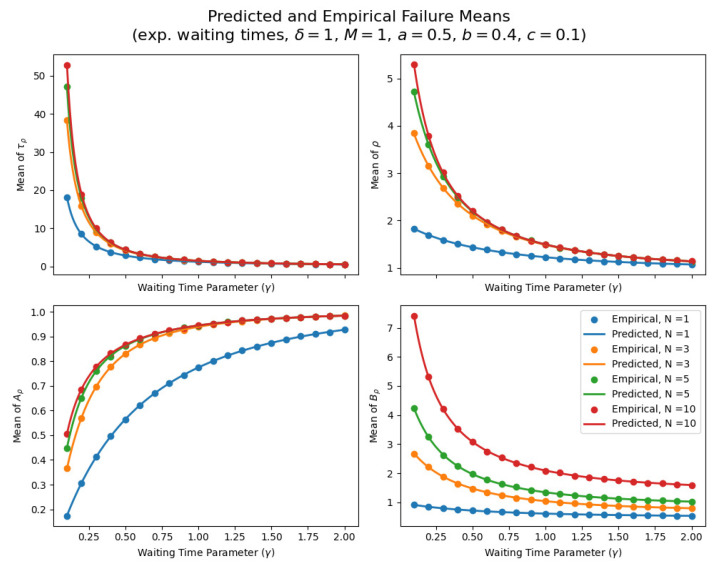
Waiting time parameter (γ) versus predicted/empirical means of ρ, $A_{\rho }$, $B_{\rho }$, and $\tau_{\rho }$.

Sample means here are based on 100,000 simulated paths for every pair (γ,N). Figure 12 below shows the predicted and estimated means of the number of shocks ρ, δ-shocks $A_{\rho }$ upon failure, (N× Extreme+Critical) shocks $B_{\rho }$, and failure time $\tau_{\rho }$. As is seen, the dots (empirical) align precisely with the means derived above and run on a much denser mesh of γ values to form smooth curves, providing additional validation.

As expected, the mean failure time $\tau_{\rho }$ always decreases as shocks become more frequent (larger γ). In addition, more frequent shocks make δ-shocks more common, so $A_{\rho }$ grows with γ. The means of ρ and $B_{\rho }$ are inversely related to γ since more frequent shocks make δ-shock failures so common, so there is a reduction in mean number of shocks at failure time and, hence, $B_{\rho }$ as well.

## 6. When the System Fails Prior to a δ-Shock

We already said in Section 4 that a single δ-shock need not necessarily ruin the system, but it can; while the occurrence of a δ-shock may not sound convincing enough to suggest the system becomes inoperational, any such event is worth checking out and so the system can be fixed if needed. We are interested in estimating the probability that the system fails through a single extreme shock or multiple critical shocks or their combination before any δ-shock takes place. Thus, we turn to functional (32)

$$\begin{array}{c} \Phi_{\mu >\nu }\left(s,y,z,\theta ,s,u,v,\vartheta ;M,N\right)=Es^{\rho }y^{A_{\rho }}z^{B_{\rho }}u^{A_{\rho -1}}v^{B_{\rho -1}}e^{-\theta \tau_{\rho }-\vartheta \tau_{\rho -1}}1_{\mu >\nu }\\ =D_{w}^{N-1}\circ D_{x}^{M-1}[\gamma_{0}\left(xy,z,\theta \right)-\gamma_{0}\left(xy,zw,\theta \right)\\ +\frac{s\gamma_{0}\left(xy,zw,\theta \right)}{1-s\gamma \left(xy,zw,\theta \right)}\left[\gamma \left(xy,z,\theta \right)-\gamma \left(xy,zw,\theta \right)\right]]\left(M,N\right) \end{array}$$

of Theorem 1 and reduce it under the same assumptions as for $\Phi_{\rho }$ made in Section 4. So, the following will be assumed:

**1**. M=1.
**2**. $\gamma_{0}=1$.
**3**. u=v=1,ϑ=0.

Hence, we arrive at the functional

(80) $$\begin{array}{c} \Phi_{\mu >\nu }\left(s,y,z,\theta ,1,1,0;1,N\right)=\varphi_{\mu >\nu }\left(s,y,z,\theta ;N\right)=Es^{\rho }y^{A_{\rho }}z^{B_{\rho }}e^{-\theta \tau_{\rho }}1_{\mu >\nu }\\ =\;D_{w}^{N-1}\circ D_{x}^{0}\left[\frac{s}{1-s\gamma \left(xy,zw,\theta \right)}\left[\gamma \left(xy,z,\theta \right)-\gamma \left(xy,zw,\theta \right)\right]\right]\\ =\;D_{w}^{N-1}\left[\frac{s\gamma \left(0,z,\theta \right)-s\gamma \left(0,zw,\theta \right)}{1-s\gamma \left(0,zw,\theta \right)}\right]\\ =\;D_{w}^{N-1}\left[\frac{1-s\gamma \left(0,zw,\theta \right)-\left[1-s\gamma \left(0,z,\theta \right)\right]}{1-s\gamma \left(0,zw,\theta \right)}\right]=D_{w}^{N-1}\left[1-\frac{1-s\gamma \left(0,z,\theta \right)}{1-s\gamma \left(0,zw,\theta \right)}\right]\\ =1-\left[1-s\gamma \left(0,z,\theta \right)\right]D_{w}^{N-1}\left[\frac{1}{1-s\gamma \left(0,zw,\theta \right)}\right]=1-\left[1-s\gamma \left(0,z,\theta \right)\right]\Pi \left(s,z,\theta \right) \end{array}$$

which is very similar to $\varphi_{\rho }$ of (56) with the same principal part $\Pi \left(s,z,\theta \right)$ obtained in (61). Note that unlike $\varphi_{\rho },$ the functional $\varphi_{\mu >\nu }$ does not depend on *y* other than that y=0 seems to be the only dependence on *y* in its right-hand side.

Thus, by purging s,y,z,θ as s=y=z=1 and θ=0, we have

$$\begin{array}{c} \varphi_{\mu >\nu }\left(1,1,1,0;N\right)=E1_{\mu >\nu }=P\left\{\nu <\mu \right\}\\ =1-\left[1-\gamma \left(0,1,0\right)\right]\Pi \left(1,1,0\right), \end{array}$$

where  $\Pi \left(1,1,0\right)=\frac{1}{\alpha +a\beta }\left[1-{\left(\frac{b\beta }{1-c\beta }\right)}^{N}\right]$, according to Formulas (4), (11) and (62)

$$\gamma \left(0,1,0\right)=\beta$$

implying that

(81) $$P\left\{\nu <\mu \right\}=1-\frac{\alpha }{\alpha +a\beta }\left[1-{\left(\frac{b\beta }{1-c\beta }\right)}^{N}\right]=\frac{a\beta }{\alpha +a\beta }+\frac{\alpha }{\alpha +a\beta }{\left(\frac{b\beta }{1-c\beta }\right)}^{N}.$$

From Section 5, we show that ${\left(\frac{b\beta }{1-c\beta }\right)}^{N}<1$. Because $\frac{\alpha }{\alpha +a\beta }$ is also less than 1, we validate that $0<P\left\{\nu <\mu \right\}<1$.

**Remark** **5** (Analysis of the system along extreme values of N and α). *From (81), we notice that $P\left\{\nu <\mu \right\}$ is monotone-decreasing in N, and it converges to $\frac{a\beta }{\alpha +a\beta }$ as N→∞ (see Remark 4 regarding N=∞). Recall that $\alpha =P\left\{\Delta <\delta \right\}$. So, the smaller is α, the larger is $P\left\{\nu <\mu \right\}$, which makes perfect sense.*

*Furthermore, for N=1 (as the critical shocks degenerate), we have that*

$$1-\frac{b\beta }{1-c\beta }=\frac{1-\left(b+c\right)\beta }{1-c\beta }=\frac{1-\left(1-a\right)\beta }{1-c\beta }=\frac{\alpha +a\beta }{1-c\beta }$$

*implying that $P\left\{\nu <\mu \right\}=1-\frac{\alpha }{1-c\beta }$.*

*Thus, we see that, under N=1, with α small and, thus β large, the probability $P\left\{\nu <\mu \right\}$ is pretty large. This is because there are no critical shocks competing with extreme shocks, as all critical and extreme shocks are just extreme shocks (as mentioned in Section 2), and the occurrence of just one extreme shock will sharply increase the likelihood of system’s failure on the basis of one extreme shock alone.*

*On the other hand, when N increases, $P\left\{\nu <\mu \right\}$ gets smaller, because now critical and extreme shocks compete, while with N large, extreme shocks, as noticed in Section 2, have an edge over critical shocks. Yet, $P\left\{\nu <\mu \right\}=\frac{a\beta }{\alpha +a\beta }$ in this case reveals an even stronger competition between extreme and δ-shocks, and with much lesser impact of the critical shocks. Note that if α is large, β is very small, making the probability $P\left\{\nu <\mu \right\}$ of an earlier failure due to one extreme shock disproportionately smaller, because β in the numerator essentially determines the value of $P\left\{\nu <\mu \right\}$.*

**Figure 13:**
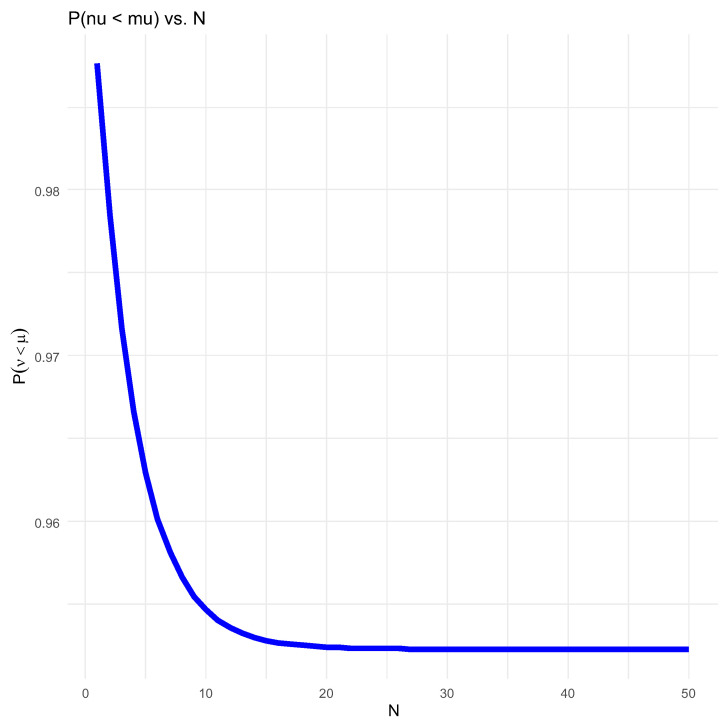
Plot of $P\left\{\nu <\mu \right\}$ as a function of *N*; for fixed $a=0.1,\;b=0.3,\;c=0.6,\;\gamma =0.1,\;\alpha =1-e^{-\gamma \delta },\;\beta =1-\alpha ,\;\delta =0.005,\;N$ varies from 1 to 50.

As noted, the graph in Figure 13 shows $P\left\{\nu <\mu \right\}\left(N\right)$ as a function decreasing in *N*.

## 7. A Modified System. Model 2. Preliminaries

In Model 2, we redefine δ-shocks to single out only those pairs of shocks with shorter time lags than δ that are either critical or extreme. In the previous sections, the δ-shocks applied to any pairs of consecutive shocks under times lags smaller than δ. The latter meant that any two consecutive harmless shocks with time lags less than δ also qualified, and because M=1, any such occurrence was deemed fatal for the system. In some models, such an occurrence is of concern. In other models, it takes more than two consecutive shocks in a row to raise flags. In the present modification, we define two consecutive shocks within a close time proximity of each other to be a threat to the system only if either of them is harmful (with some further constraints to follow). Note that in the event two consecutive harmful shocks occur within a time frame less than δ, there can be arbitrarily many harmless shocks in between, of which all were δ-shocks in the context of Model 1. Now this is no longer the case.

An extreme shock can be a δ-shock but only if it is preceded by a critical shock. In this case, the system fails on two counts. If an extreme shock is not δ, the system instantly fails without giving a chance to any consecutive harmful shock to be δ. Thus, a harmful shock can be δ only if it is a consecutive shock. Consequently, it can be critical (in particular, *N*th critical) or extreme.

In a nutshell, in a pair of two consecutive harmful shocks with a time lag less than δ, the second shock is deemed a δ-shock if the first of the two is neither extreme nor *N*th critical.

As mentioned, the harmless shocks still land in the system, but they are no longer counted as δ-shocks regardless of how many of them occur consecutively with time lags less than δ.

In contrast with Model 1, we assume that at time $\tau_{0},$ when the system was first observed, exactly one, strictly critical, shock landed (that is, at any time $t\leq \tau_{0}$).

We form the process of harmful shocks from $\left\{\tau_{n}\right\}$. Suppose $T_{1}$ is the time of the first harmful shock after epoch $\tau_{0}$, that is,

(82) $$T_{1}=\min\left\{\tau_{n}:n\in N:W_{n}>H_{1}\right\}$$

and furthermore,

(83) $$T_{j}=\min\left\{\tau_{n}>T_{j-1}:W_{n}>H_{1}\right\},j=1,2,\ldots ,T_{0}=\tau_{0}=0.$$

Thus, $\left\{T_{j}\right\}$ is an embedded sequence of consecutive harmful shocks (that excludes harmless shocks).

We proceed with a more rigorous construction of the embedded point process $\left\{T_{j}\right\}$. Define the random index

(84) $$\eta =\eta_{1}=\min\left\{n\in N:W_{n}>H_{1}\right\}.$$

Then, $T_{1}=\tau_{\eta }$ and furthermore,

(85) $$\eta_{j}=\min\left\{n>\eta_{j-1}:W_{n}>H_{1}\right\},\;j=2,3,\ldots$$

implying that

(86) $$T_{j}=\tau_{\eta_{j}},\;j=0,1,\ldots ,\;\mathrm{with}\;\eta_{0}=0.$$

**Figure 14:**
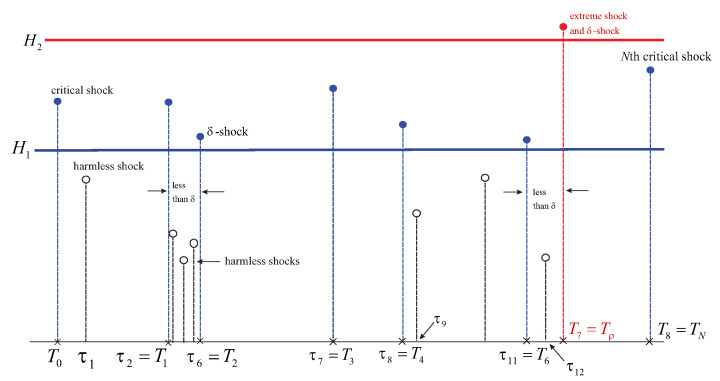
A System where δ-shocks can only be among harmful shocks (i.e., critical or extreme).

In Figure 14, below, we focus on new variants of δ-shocks. Here, we see a path of the shocks process in which δ-shocks can only be among harmful shocks. In particular, $\tau_{6}=T_{2}$ is identified as the second critical shock and also a δ-shock. The three other shocks squeezed between $T_{1}=\tau_{2}$ and $T_{2}=\tau_{6}$ are harmless, and their roles reduce only to the determination of the distance between consecutive harmful shocks (critical or extreme). Thus, the δ-shock at $\tau_{6}=T_{2}$ is also fatal.

In another scenario, for convenience depicted in the same figure, we assume that the shock at $T_{2}$ is not δ. Then, the system will keep functioning until eventually reaching the time-to-failure at $T_{\rho }$ (introduced in Section 8), that is, at $T_{7}$ when the first extreme critical shock lands. This shock becomes fatal on two counts: firstly, because it is extreme, and secondly, because it is also δ. If neither of these were to take place at $T_{7},$ then the next harmful shock would be fatal, because it is *N*th critical (assuming that N=8).

It seems obvious that $\sum_{k=0}^{\infty }\varepsilon_{T_{k}}$ is a delayed renewal process of consecutive harmful shocks that we will mark in a few moments. The “delay” is driven by one critical shock striking the system at time $T_{0}$ or earlier but associated with $T_{0}$.

Marked point process of shocks: To identify δ-shocks we start with the sequence of i.i.d. Bernoulli r.v.s

(87) $$V_{j}=1_{\left\{T_{j}-T_{j-1}<\delta \right\}},j=1,2,\ldots$$

(being identifiers of δ-shocks) followed by another sequence

(88) $$U_{j}=N1_{\left\{W_{\eta_{j}}>H_{2}\right\}}+1_{\left\{H_{1}<W_{\eta_{j}}\leq H_{2}\right\}}$$

of identifiers as i.i.d. binary r.v.s valued in $\left\{1,N\right\}$. Because $W_{\eta_{j}}>H_{1}$ a.s., there is no need to include the number 0 in the set $\left\{1,N\right\}$ as much as any other number equally irrelevant.

With the shocks identifiers $\left(V_{j},U_{j}\right),$ we complete our marking of the point process $\sum_{k=0}^{\infty }\varepsilon_{T_{k}}$ (now the support counting measure) as a delayed marked renewal process

(89) $$\left(V,U,T\right)=\sum_{k=0}^{\infty }\left(V_{j},U_{j}\right)\varepsilon_{T_{k}}.$$

Note that $\left(V,U,T\right)$ runs indefinitely, continually hitting the system even after it fails. We fix it in Section 8 after some more formalism.

For the forthcoming analysis, we need to find the joint functional

(90) $$\Gamma \left(y,z,\theta \right)=Ey^{V}z^{U}e^{-\theta \tau_{\eta }}=Ey^{V}z^{U}e^{-\theta T_{1}},$$

where $\left[V\right]$ and $\left[U\right]$ are associated equivalence classes of r.v.s distributed as $V_{j}$s and $U_{j}$s, respectively, and $\tau_{\eta }$ is the time between $T_{0}=\tau_{0}$ and $T_{1}$.

We begin with the marginal functional $\Gamma \left(1,z,\theta \right)=Ez^{U}e^{-\theta \tau_{\eta }}$, which satisfies the key fluctuation theorem (Dshalalow [35]) established there for a marked delayed renewal process with three active components and holding also for a single active component, in this case *U*. A component in a multivariate marked process is deemed active if it is supposed to cross some critical threshold. Any other component that has no threshold to cross is referred to as passive. If a multivariate marked point process carries only one active component, say, U, all other passive components assume their respective values on *U*’s crossing. For example, if another passive component is a time component, then it registers the time when *U* crosses that threshold. All other passive components assume their values accordingly at the time of *U*’s crossing. The process no longer evolves after this event, or the rest of its future is of no further interest.

If a multivariate marked process has more than one active component, there is a competition (or a game) between them, in which one of the active components hits their associated threshold first. When it occurs, the rest of active as well as all passive components assume their respective values, and the process stops. We dealt with this situation in Theorem 1, established specifically for a wide class of reliability models with competing failure processes.

Now, of the two components *U* and $T_{1}=\tau_{\eta }$ in $\Gamma \left(1,z,\theta \right)=Ez^{U}e^{-\theta \tau_{\eta }},$$\tau_{\eta }$ is passive and it assumes its time value when *U* turns 1 or *N* for the first time after $T_{0}$. To apply the key fluctuation theorem, we first turn to Section 2 concerning the functional $\gamma \left(y,z,\theta \right)=Ey^{X}z^{Y}e^{-\theta \Delta },$ although we focus on the two last components, *Y* and Δ. Recall that *Y* took values 0,1,N, but from the above setting we are interested in the binary version of *Y* when *Y* is either 0 or greater than 0.

Recall that in Section 2, the sequence $\left\{B_{n}\right\}$ of partial sums $B_{n}=\sum_{k=1}^{n}Y_{k}$ was associated with index ν=min$\left\{n:B_{n}\geq N\right\},$ which would have been a ruin index in the absence of δ-shocks. This was because the system (with no δ-shocks) was harassed exclusively by harmless, critical, and extreme shocks, and because the system could endure some number of critical shocks and one extreme shock to land at the total of more than *N* shocks altogether upon its failure. In our present setting, we deal with a special case when the process of shocks is “suspended” or, rather, observed at $\tau_{\eta }$ when the first harmful shock lands, which can be either critical or extreme and thus valued 1 or N, respectively.

To make use of the key fluctuation theorem, we temporarily dismiss the initial critical shock at $\tau_{0}$ and set ν=min$\left\{n\in N:B_{n}\geq 1\right\}$. The suspension of the initial critical shock makes us assume that $Y_{0}=B_{0}=0$. Correspondingly, if $B_{n}$ turns ≥1 for some n>0 at the first time, it means that $B_{n-1}=0$ and so are all other *B*s with lower indices, but $B_{n}\in \left\{1,N\right\}$. In the event the more general version of ν=min$\left\{n:B_{n}\geq N\right\}$ is of interest, we would use the formula

(91) $$\Gamma \left(1,z,\theta \right)=1-\left[1-\gamma \left(1,z,\theta \right)\right]D_{x}^{N-1}\frac{1}{1-\gamma \left(1,zx,\theta \right)}$$

as per Dshalalow [35] (or even Dshalalow’s earlier results pertaining to this basic case). Operator D is the same as the one in $\left(Di\right)$ of Section 3. In our present case, as argued, we need the version of (91) precisely for N=1, namely,

$$\Gamma \left(1,z,\theta \right)=1-\left[1-\gamma \left(1,z,\theta \right)\right]D_{x}^{0}\frac{1}{1-\gamma \left(1,zx,\theta \right)}$$

that instantly reduces the right-hand side of (91) to

(92) $$\Gamma \left(1,z,\theta \right)=1-\left[1-\gamma \left(1,z,\theta \right)\right]\frac{1}{1-\gamma \left(1,0,\theta \right)}$$

as per $\left(Di\right)$. Note that with all the simplicity of (92), the formula would be difficult to deduce by direct probabilistic means.

Now recall from (15) that under the assumed independence of r.v.s *Y* (an integer-valued identifier of W) and Δ,

(93) $$\gamma \left(1,z,\theta \right)=a\left(z\right)g\left(1,\theta \right)=\left[az^{N}+bz+c\right]\gamma \left(\theta \right),$$

with

(94) $$\gamma \left(\theta \right)=\gamma \left(1,1,\theta \right)=Ee^{-\theta \Delta },$$

while

(95) $$\gamma \left(1,0,\theta \right)=c\gamma \left(\theta \right)$$

needed in (92).

Substituting (93)–(95) into (92) yields

(96) $$\begin{array}{c} \Gamma \left(1,z,\theta \right)=1-\left[1-\left[az^{N}+bz+c\right]\gamma \left(\theta \right)\right]\frac{1}{1-c\gamma \left(\theta \right)}\\ =1-\frac{1-a\left(z\right)\gamma \left(\theta \right)}{1-c\gamma \left(\theta \right)}=\frac{1-c\gamma \left(\theta \right)-1+a\left(z\right)\gamma \left(\theta \right)}{1-c\gamma \left(\theta \right)}=\gamma \left(\theta \right)\frac{a\left(z\right)-c}{1-c\gamma \left(\theta \right)}=\gamma \left(\theta \right)\frac{az^{N}+bz}{1-c\gamma \left(\theta \right)}\\ =\gamma \left(\theta \right)\frac{a_{0}z^{N}+b_{0}z}{\frac{1}{p}-\left(1-p\right)\frac{1}{p}\gamma \left(\theta \right)}=a_{0}\left(z\right)\frac{p\gamma \left(\theta \right)}{1-\left(1-p\right)\gamma \left(\theta \right)}, \end{array}$$

where

(97) $$p=a+b,\;a_{0}=\frac{a}{a+b},\;b_{0}=\frac{b}{a+b},\;a_{0}\left(z\right)=a_{0}z^{N}+b_{0}z=\frac{1}{p}\left[az^{N}+bz\right].$$

**Remark** **6.** *Formulas (96) and (97) embellish the marginal distribution*

(98) $$\Gamma \left(\theta \right)=\Gamma \left(1,1,\theta \right)=Ee^{-\theta \tau_{\eta }}=\frac{p\gamma \left(\theta \right)}{1-q\gamma \left(\theta \right)},\;\mathrm{with}\;E\tau_{\eta }=\frac{1}{p\gamma }.$$

*(which is type 1 geometric with interrenewal times included in the classic geometric experiment of a series of independent Bernoulli trials) that alone could be readily obtained by the double expectation formula without the use of fluctuation calculus. However, the joint distribution $\Gamma \left(1,z,\theta \right)$ is more difficult to justify using straightforward probability arguments. Furthermore, the factor $a_{0}\left(z\right)$ in (96) and (97) points to a rather surprising outcome that the r.v.s U and $\tau_{\eta }$ are independent, which would not be obvious when using other means. Furthermore, Formula (96) identifies the distribution of r.v. U that looks conditioned on set $\Omega_{0}=E_{2}\cup E_{3}=\left\{W>H_{1}\right\}$, implying that*

$$a_{0}\left(z\right)=E\left[z^{Y}|\Omega_{0}\right]=\frac{1}{P\left(\Omega_{0}\right)}E\left[z^{Y}1_{\Omega_{0}}\right].$$

*In a nutshell, fluctuation calculus turns out to be a straightforward method that gives a fully secure result, circumventing common ambiguities of the double expectation (in some difficult cases) and other, less conventional, tools.*

Thus, $\left\{T_{n}\right\}$ is an embedded point process with the marginal LST of the interrenewal times satisfying Formula (98). In particular, if $\Delta \in \left[\mathrm{Exp}\left(\gamma \right)\right],$ that is, when $\gamma \left(\theta \right)=\frac{\gamma }{\gamma +\theta },\;$

(99) $$\Gamma \left(\theta \right)=Ee^{-\theta T_{1}}=\frac{p\gamma }{p\gamma +\theta },\;i.e.,\;T_{1}\in \left[\mathrm{Exp}\left(p\gamma \right)\right].$$

In conclusion, we consider a modified system with shocks landing at $T_{0}=0,T_{1},T_{2},\ldots ,$ of magnitudes $W_{0},W_{1},W_{2},\ldots$ such that $H_{1}<W_{0}\leq H_{2}$; for the other *W*s, when $H_{1}<W_{i}\leq H_{2},$ the shock at $T_{i}$ is critical, and when $W_{i}>H_{2},$ the shock is extreme and thus fatal. The system fails if a single extreme shock hits the system at some time $T_{i}$ or if a shock at $T_{i}$ is *N*th critical, counting from that at $T_{0}$. To avoid triviality, we thus assume that N>1. The δ-policy has not been introduced yet.

The former δ-shock policy applied to any types or mixes of shocks is altered in the following way. It is now restricted entirely to critical or extreme shocks (harmful shocks). More specifically, if a shock that landed at time $T_{i},$ is such that $\left|(T_{i-1},T_{i}]\right|<\delta ,$ this shock is referred to as a δ-shock, provided that the shock at $T_{i-1}$ is critical but not *N*th critical. That said, the shock at $T_{i}$ can be $\left(a\right)$ critical, $\left(b\right)$*N*th critical, $\left(c\right)$ extreme.

Now we are back to the formalism of functional $\Gamma \left(y,z,\theta \right)=Ey^{V}z^{U}e^{-\theta \tau_{\eta }},$ where $V=1_{\left\{\tau_{\eta }<\delta \right\}}$. This functional was not a part of the key fluctuation formula, because combined with V, the underlying trivariate process did not meet the conditions in the associated theorem of [35]. However, with the newly established *U* that turned independent from $\tau_{\eta },$ we can use the same argument as in the formation of $\gamma \left(y,z,\theta \right)$ regarding *U* and $\left(V,\tau_{\eta }\right)$ as independent. Thus, because *V* is binary with

$$y^{V}=y^{1_{\left\{\tau_{\eta }<\delta \right\}}}=y1_{\left\{\tau_{\eta }<\delta \right\}}+1_{\left\{\tau_{\eta }\geq \delta \right\}},$$

we define $G\left(y,\theta \right)$ as the marginal of $\Gamma \left(y,z,\theta \right)$ in the form

(100) $$\begin{array}{cc} G\left(y,\theta \right) & =Ee^{-\theta \tau_{\eta }}y^{V}=yEe^{-\theta \tau_{\eta }}1_{\left\{\tau_{\eta }<\delta \right\}}+Ee^{-\theta \tau_{\eta }}1_{\left\{\tau_{\eta }\geq \delta \right\}}\\ \; & =\alpha_{0}\left(\theta \right)y+\beta_{0}\left(\theta \right),\; \end{array}$$

with

(101) $$\alpha_{0}\left(\theta \right)=Ee^{-\theta \tau_{\eta }}1_{\left\{\tau_{\eta }<\delta \right\}}\;\mathrm{and}\;\beta_{0}\left(\theta \right)=Ee^{-\theta \tau_{\eta }}1_{\left\{\tau_{\eta }\geq \delta \right\}.}$$

Note that

$$\alpha_{0}\left(\theta \right)+\beta_{0}\left(\theta \right)=Ee^{-\theta \tau_{\eta }}=\Gamma \left(\theta \right)=\Gamma \left(1,1,\theta \right)=G\left(1,\theta \right)\;$$

was attached to $\Gamma \left(1,z,\theta \right)=a_{0}\left(z\right)\Gamma \left(\theta \right)$$\left[\mathrm{with}\;\Gamma \left(\theta \right)=\frac{p\gamma \left(\theta \right)}{1-\left(1-p\right)\gamma \left(\theta \right)}\right]$.

Now replacing $\Gamma \left(\theta \right)$ in the latter expression with $G\left(y,\theta \right),$ we come to specify $\Gamma \left(y,z,\theta \right)=Ey^{V}z^{U}e^{-\theta \tau_{\eta }}$ as

(102) $$\Gamma \left(y,z,\theta \right)=a_{0}\left(z\right)G\left(y,\theta \right)=\left(a_{0}z^{N}+b_{0}z\right)\left[\alpha_{0}\left(\theta \right)y+\beta_{0}\left(\theta \right)\right].$$

Note that $\alpha_{0}\left(\theta \right)$ and $\beta_{0}\left(\theta \right)$ in (101) are implicit unless we specify them as in our forthcoming discussion in Example 3.

Finally,

(103) $$\alpha_{0}=\alpha_{0}\left(0\right)=P\left\{\tau_{\eta }<\delta \right\}\;\mathrm{and}\;\beta_{0}=\beta_{0}\left(0\right)=P\left\{\tau_{\eta }\geq \delta \right\},\;\mathrm{with}\;\alpha_{0}+\beta_{0}=1$$

where $\left(\alpha_{0},\beta_{0}\right)$ is the marginal distribution of V, with the PGF

(104) $$Ey^{V}=G\left(y,0\right)=\alpha_{0}y+\beta_{0}.$$

In summary, we note the following:

**Proposition** **1.** *In Model 2, where δ-shocks are formed through pairs of consecutive harmful shocks with time lags less than δ, the associated marked point process of harmful shocks (embedded in the process of all shocks of Model 1) is a marked delayed renewal process, with interrenewal times, jointly with their marks Us and Vs, and are distributed in accordance with the functional $\Gamma \left(y,z,\theta \right)$, satisfying formula (102) and exhibiting independence of U and $\left(V,\tau_{\eta }\right)$ with respective marginal transforms in (100)–(104).*

The distribution of the delay is unspecified and so far is arbitrary.

Note that we have not restricted δ-shocks as to how they turn fatal (which we do in the forthcoming sections), nor did we specify exactly how the system fails, except for some allusions and loose preliminaries.

**Remark** **7** (An informal discussion). *Assume we have a process of shocks reduced to harmful shocks only, thus with one threshold $H_{2}$. Any shock with a magnitude below $H_{2}$ is critical and above $H_{2}$ is extreme. Suppose the associated marked random measure is delayed renewal with assumed position-independent marking. The above specifications of U,V, and $T\;\;(=\tau_{\eta })$ apply but with the distribution of T being arbitrary. The conditions are the same as in Proposition 1, except that the position independence is now assumed rather than proved. Furthermore, Proposition 1 yields the special case $\Gamma \left(1,1,\theta \right)=\frac{p\gamma \left(\theta \right)}{1-\left(1-p\right)\gamma \left(\theta \right)}$ of the marginal functional $\Gamma \left(y,z,\theta \right)$ instead of no assumption on $\Gamma \left(\cdot \right)$. Furthermore, Proposition 1 suggests that $T_{1},T_{2},\ldots$ are the successive epochs of harmful shocks and thus with independent and identically distributed interarrival times following the principles of a “geometric process” of some arrivals at random epochs of time until the first success, with $\Gamma \left(1,1,\theta \right)=\frac{p\gamma \left(\theta \right)}{1-\left(1-p\right)\gamma \left(\theta \right)}$ using the double expectation formula.*

*Then we used the key fluctuation theorem to arrive at $\Gamma \left(1,z,\theta \right)=a_{0}\left(z\right)\frac{p\gamma \left(\theta \right)}{1-\left(1-p\right)\gamma \left(\theta \right)}$, where $a_{0}\left(z\right)$ is the new marginal of shocks’ binary identifiers conditioned on $\Omega_{0}$ that they are exclusively harmful. The consequence altogether is that under the above actions, we are now on the new traced probability space $\left(\Omega_{0},F\cap \Omega_{0},P_{0}\right),$ $P_{0}=P\left(\cdot \cap \Omega_{0}\right)/P\left(\Omega_{0}\right),$ where there is no place for harmless shocks anymore. See more in Section 8.*

## 8. Further Formalism of Model 2

**Remark** **8.** *Reiterating what was said in Remark 7, we note that while in Section 2, $Y\in \left\{N,1,0\right\}$ with the respective distribution $\left\{a,b,c\right\},$ the associated identifier U is valued in $\left\{N,1\right\}$ under the distribution $\left\{a_{0},b_{0},\right\}$, as per (97).*

*With $\Omega_{0}=\left\{W>H_{1}\right\}=\left\{Y\in \left\{1,N\right\}\right\}=E_{2}\cup E_{3},$ the above marginal PGF $a_{0}\left(z\right)$ of U in (97) can also be justified using the conditional expectation:*

$$\begin{array}{cc} a_{0}\left(z\right) & =E\left[z^{Y}|\Omega_{0}\right]=E\left[z^{Y}1_{\Omega_{0}}\right]\frac{1}{P\left(\Omega_{0}\right)}=\frac{1}{P\left(\Omega_{0}\right)}\int_{\Omega_{0}}z^{Y}dP=\frac{1}{P\left(\Omega_{0}\right)}\int_{y\in \left\{N,1\right\}}z^{y}P_{Y}\left(dy\right)\\ \; & =\frac{1}{a+b}\left[z^{N}P\left\{Y=N\right\}+zP\left\{Y=1\right\}\right]=\frac{1}{a+b}\left[az^{N}+bz\right]\\ \; & =a_{0}z^{N}+b_{0}z, \end{array}$$

*or as $E_{0}\left(\cdot \right)=E\left[\left(\cdot \right)1_{\Omega_{0}}\right]\frac{1}{P\left(\Omega_{0}\right)}=\frac{1}{P\left(\Omega_{0}\right)}\int_{\omega \in \Omega_{0}}\left(\cdot \right)P\left(d\omega \right)$, which is the associated expectation relative to the traced probability space $\left(\Omega_{0},F\cap \Omega_{0},P_{0}\right)$. Here, $P_{0}$ is the conditional probability measure $E\left[\left(\cdot \right)1_{\Omega_{0}}\right]\frac{1}{P\left(\Omega_{0}\right)}$. We will, however, relax the measure-theoretical contents of our forthcoming calculus.*

*For notational convenience, we will use P for the conditional probability measure $\left(P_{0}\right)$ and the associated conditional expectation as E$\left(\mathrm{in}\;\mathrm{place}\;\mathrm{of}\;E_{0}\right)$, bearing in mind, however, that we deal with the system on the traced space, in which the harmless shocks play no role beyond the determination of the joint distribution of the times between consecutive harmful (critical or extreme) shocks and the associated shocks identifiers.*

Consequently, the embedded process of shocks can be seen upon $T_{1},T_{2}\ldots$ through $U_{1},U_{2},\ldots ,$ where $U_{k}\in \left[U\right],k=1,2,\ldots$ Of course, *U*s are shock identifiers and they represent the respective magnitudes of shocks $W_{\eta_{1}},W_{\eta_{2}},\ldots$ at $T_{1},T_{2},\ldots$ which can now only be critical or extreme. Hence, the associated embedded marked point process of times and shock magnitudes $\sum_{k=1}^{\infty }W_{\eta_{k}}\varepsilon_{T_{k}}$ can be replaced with a cruder but sufficiently descriptive variant $\sum_{k=1}^{\infty }U_{k}\varepsilon_{T_{k}}$ that will be better suited for the associated random walk analysis that proceeds under the same course as in Section 2 and Section 3, starting with

(105) $$U_{n}=\sum_{k=1}^{n}U_{k},\;n=1,2,\ldots ,$$

forming the sequence of partial sums of $\left\{U_{k}\right\}$ with

(106) $$\zeta =min\left\{n:U_{n}\geq N\right\}.$$

The δ-shocks are included in (89) via the sequence of i.i.d. Bernoulli r.v.s

(107) $$V_{k}=1_{\left\{T_{k}-T_{k-1}<\delta \right\}},k=1,2,\ldots ,T_{0}=0,$$

Acting alone, the sequence would continue until $V_{k}=1$ a.s. However, the sequence as well as the whole process $\left(V,U,T\right)=\sum_{k=0}^{\infty }\left(V_{j},U_{j}\right)\varepsilon_{T_{k}}$ can be interrupted by an earlier occurrence of an extreme or *N*th critical shock.

Now we define

(108) $$\chi =min\{m:A_{m}=\sum_{k=1}^{m}V_{k}=M\}\;\mathrm{for}\;\;\mathrm{some}\;M\geq 1,$$

tentatively assuming that *M* δ-shocks occurring in any order will ruin the system at time $T_{\chi }$ unless other harmful shocks will cause an earlier failure. We will again deal only with the special case M=1, although Theorem 1 is formulated for the general value of *M* (that we plan to explore in our forthcoming paper). The cumulative ruin index is then

(109) ρ=ζ∧χ.

(while it would be more proper to use some different character for ζ∧χ than ρ to tell it from ρ in Section 2, Section 3, Section 4, Section 5 and Section 6, it would be harder to associate it with the common ρ in Theorem 1).

Consequently, $T_{\rho }$ is the time-to-failure of this system. Under this formalism of $T_{\rho },$ we can revisit Figure 14 and the preceding interpretation, which now makes more sense.

Analogous to Section 2, denote $\left[\tau_{\eta }\right]$ as the equivalence class of all r.v.s having the same distribution as $T_{i}-T_{i-1},i=1,2,\ldots$. Then, the failure time of the system occurs at $T_{\rho },$ with the total count of critical and extreme shocks $U_{\rho }$ and δ-shocks count $A_{\rho }\;$ the on system’s failure. Consequently, the marked process $\left(V,U,T\right)=\sum_{k=0}^{\infty }\left(V_{j},U_{j}\right)\varepsilon_{T_{k}}$ of (89) is to be curtailed to

$${\left(V,U,T\right)}_{\rho }=\sum_{k=0}^{\rho }\left(V_{j},U_{j}\right)\varepsilon_{T_{k}},$$

that is, until it ends at $T_{\rho }$.

**Example** **3.** *We revisit Example 1 in a similar context. Recall that back then, we set the* Δ-*marginal distribution of $\left(X,\Delta \right)$ exponential with parameter γ, that is, $\Delta \in \left[\mathrm{Exp}\left(\gamma \right)\right]$. This assumption as we pointed out in Section 7 implied that $\tau_{\eta }\in \left[\mathrm{Exp}\left(\gamma p\right)\right],$ where p=a+b. Now it takes very little to adjust all computations in Example 1 replacing γ with γp. Yet we proceed with details under the new notation:*

(110) $$\alpha_{0}\left(\theta \right)=Ee^{-\theta \tau_{\eta }}1_{\left\{\tau_{\eta }<\delta \right\}}=\gamma p\int_{x=0}^{\delta }e^{-(\theta +\gamma p)x}dx=\frac{\gamma p}{\gamma p+\theta }\left[1-e^{-\left(\gamma p+\theta \right)\delta }\right]$$

*and*

(111) $$\beta_{0}\left(\theta \right)=E1_{\left\{\tau_{\eta }\geq \delta \right\}}e^{-\theta \tau_{\eta }}=\frac{\gamma p}{\gamma p+\theta }-\frac{\gamma p}{\gamma p+\theta }\left[1-e^{-\left(\gamma p+\theta \right)\delta }\right]=\frac{\gamma p}{\gamma p+\theta }e^{-\left(\gamma p+\theta \right)\delta }.$$

*Then, the marginal distribution of V is*

(112) $$\alpha_{0}=\alpha_{0}\left(0\right)=1-e^{-\gamma p\delta }\;and\;\beta_{0}=\beta_{0}\left(0\right)=e^{-\gamma p\delta }.$$

*Hence,*

(113) $$\begin{array}{cc} G\left(y,\theta \right) & =Ey^{V}e^{-\theta \tau_{\eta }}=Ey^{1_{\left\{\tau_{\eta }<\delta \right\}}}e^{-\theta \tau_{\eta }}=\alpha_{0}\left(\theta \right)y+\beta_{0}\left(\theta \right)\\ \; & =\frac{\gamma p}{\gamma p+\theta }\left[y\left(1-e^{-\left(\gamma p+\theta \right)\delta }\right)+e^{-\left(\gamma p+\theta \right)\delta }\right]. \end{array}$$

*Now, from (113),*

(114) $$\frac{\partial }{\partial y}G\left(y,\theta \right){|}_{y=1}=\frac{\gamma p}{\gamma p+\theta }\left(1-e^{-\gamma p\delta }e^{-\theta \delta }\right),$$

*and further from (114) we obtain*

$$R\left(\tau_{\eta },V\right)=E\left[\tau_{\eta }\cdot V_{\eta }\right]=\left(-1\right)\frac{d}{d\theta }G\left(1,\theta \right){|}_{\theta =0}=\frac{1}{\gamma p}\left(1-e^{-\gamma p\delta }\right)+\delta e^{-\gamma p\delta }.$$

*Therefore, from the last expression, (112), and that $E\tau_{\eta }=\frac{1}{\gamma p},$*

(115) $$Cov\left(\tau_{\eta },V\right)=\frac{1}{\gamma p}\left(1-e^{-\gamma p\delta }\right)+\delta e^{-\gamma p\delta }-\frac{1}{\gamma p}\left(1-e^{-\gamma p\delta }\right)=\delta e^{-\gamma p\delta }.$$

## 9. Competing Processes

Since the new system is similar to that treated in Section 2, Section 3, Section 4, Section 5 and Section 6, we abridge our reasoning and computations making only some necessary adjustments. The formula analogous to (52) reads

(116) $$\begin{array}{cc} \Phi_{\rho }\left(s,y,z,\theta ,1,1,0;M,N\right) & =Es^{\rho }y^{A_{\rho }}z^{B_{\rho }}e^{-\theta T_{\rho }}\\ \; & =D_{w}^{N-1}\circ D_{x}^{M-1}[\Gamma_{0}\left(y,z,\theta \right)-\Gamma_{0}\left(xy,zw,\theta \right)\\ \; & +\frac{s\Gamma_{0}\left(xy,zw,\theta \right)}{1-s\Gamma \left(xy,zw,\theta \right)}\left[\Gamma \left(y,z,\theta \right)-\Gamma \left(xy,zw,\theta \right)\right]]. \end{array}$$

Here, M≥1 but it will be reduced to M=1, while now N≥2, because we assumed that the system started with one critical shock that landed at $T_{0}=0.\;$ Thus,

$$\Gamma_{0}\left(y,z,\theta \right)=Ey^{A_{0}}z^{V_{0}}e^{-\theta T_{0}}=z,$$

because $A_{0}=V_{0}=T_{0}=0,$ while $U_{0}=1$ a.s. as previously defined. With no restriction on *N*, rather than N≥2, we now set M=1, implying that

(117) $$\begin{array}{cc} \psi_{\rho }\left(s,y,z,\theta ;N\right) & =Es^{\rho }y^{A_{\rho }}z^{B_{\rho }}e^{-\theta T_{\rho }}\\ & =D_{w}^{N-1}\circ D_{x}^{0}\left[z-zw+\frac{szw}{1-s\Gamma \left(xy,zw,\theta \right)}\left[\Gamma \left(y,z,\theta \right)-\Gamma \left(xy,zw,\theta \right)\right]\right]\\ & =D_{w}^{N-1}\left[z-zw+\frac{szw\left[\Gamma \left(y,z,\theta \right)-\Gamma \left(0,zw,\theta \right)\right]}{1-s\Gamma \left(0,zw,\theta \right)}\right]=z-z+zD_{w}^{N-2}\frac{s\Gamma \left(y,z,\theta \right)-1+1-s\Gamma \left(0,zw,\theta \right)}{1-s\Gamma \left(0,zw,\theta \right)}\\ & =0+zD_{w}^{N-2}\left[1-\frac{1-s\Gamma \left(y,z,\theta \right)}{1-s\Gamma \left(0,zw,\theta \right)}\right]=z-z\left[1-s\Gamma \left(y,z,\theta \right)\right]D_{w}^{N-2}\frac{1}{1-s\Gamma \left(0,zw,\theta \right)}. \end{array}$$

**Remark** **9.** *In particular, the marginal transform of $T_{\rho }$ turns*

(117a) $$\psi_{\rho }\left(1,1,1,\theta ;N\right)=Ee^{-\theta T_{\rho }}=1-\left[1-\Gamma \left(1,1,\theta \right)\right]D_{w}^{N-2}\frac{1}{1-\Gamma \left(0,w,\theta \right)}$$

*with*

(117b) $$ET_{\rho }=-\frac{\partial }{\partial \theta }\Gamma \left(1,1,\theta \right){|}_{\theta =0}D_{w}^{N-2}\frac{1}{1-\Gamma \left(0,w,0\right)},$$

*where*

(117c) $$-\frac{\partial }{\partial \theta }\Gamma \left(1,1,\theta \right){|}_{\theta =0}=E\tau_{\eta }$$

*and [readily from (117)]*

(117d) $$D_{w}^{N-2}\frac{1}{1-\Gamma \left(0,w,0\right)}=E\rho =\Pi \left(1,1,0\right).$$

*The latter is the mean value of the total count ρ (if we are still dealing with shocks, although the above Formula (117) is for an unspecified process) of all harmful shocks until failure. Indeed, the ρ-marginal PGF is*

(117e) $$Es^{\rho }=Es^{\rho }y^{A_{\rho }}z^{B_{\rho }}e^{-\theta T_{\rho }}{|}_{y=z=1,\theta =0}=1+\left[s-1\right]D_{w}^{N-2}\frac{1}{1-s\Gamma \left(0,w,0\right)}$$

*implying that*

(117f) $$E\rho =\Pi \left(1,1,0\right)=D_{w}^{N-2}\frac{1}{1-\Gamma \left(0,w,0\right)}.$$

*In conclusion,*

(117g) $$ET_{\rho }=E\tau_{\eta }\cdot E\rho =ET_{1}\cdot E\rho .$$

*This formula holds without any special assumptions or specifications rendered in Section 7 and Section 8. It is even invariant of any interpretation imposed on the process dealt with in (117).*

Returning to Formula (117), note that it looks similar to (55).They differ in factor *z* and in $\Gamma \left(\cdot \right)$ in (117), replacing $\gamma \left(\cdot \right)$ in (55).

Returning to the special case pertaining to Model 2 specified in Section 7, from

$$\Gamma \left(y,z,\theta \right)=a_{0}\left(z\right)G\left(y,\theta \right)=\left[a_{0}z^{N}+b_{0}z\right]\left(\alpha_{0}\left(\theta \right)y+\beta_{0}\left(\theta \right)\right],$$

we write down expression $\frac{1}{1-s\Gamma \left(0,zw,\theta \right)}$ in (117) in its explicit form as

$$F\left(s,zw,\theta \right)=\frac{1}{1-s\Gamma \left(0,zw,\theta \right)}=\frac{1}{1-s\left[a_{0}z^{N}w^{N}+b_{0}zw\right]\beta_{0}\left(\theta \right)}=\frac{1}{1-\left[A_{0}w^{N}+B_{0}w\right]},$$

where $A_{0}=sa_{0}\beta_{0}\left(\theta \right)z^{N},B_{0}=sb_{0}\beta_{0}\left(\theta \right)z$. *F* looks simpler than its counterpart in (57). This is because its polynomial in the denominator does not carry a constant (57) has.

Expanding $F\left(s,zw,\theta \right)$ in series of powers of $A_{0}w^{N}+B_{0}w$ gives

$$S\left(s,zw,\theta \right)=\sum_{n=0}^{\infty }{\left[A_{0}w^{N}+B_{0}w\right]}^{n}=\sum_{n=0}^{\infty }{\left[A_{0}w^{N-1}+B_{0}\right]}^{n}w^{n}.$$

Series *S* converges to *F* in a vicinity of w=0. Then we apply operator $D_{w}^{N-2}$ to *S* to obtain

$$\begin{array}{cc} \Pi \left(s,z,\theta \right) & =D_{w}^{N-2}F\left(s,zw,\theta \right)=D_{w}^{N-2}S\left(s,zw,\theta \right)\\ \; & =\sum_{n=0}^{N-2}D_{w}^{N-2-n}{\left[A_{0}w^{N-1}+B_{0}\right]}^{n}=\sum_{n=0}^{N-2}B_{0}^{n}. \end{array}$$

Now we need the following:

**Lemma** **2.** *For n=0,1,…R, it holds true that*

$$D_{w}^{R-n}{\left(aw^{R+1}+b\right)}^{n}=b^{n}.\left(HereR=N-2.\right)$$

**Proof.**  Firstly,

DwR−nawR+1+bn=∑k=0nnkakbn−kDwR−nw(R+1)k.

Then, for $k=0,\;D_{w}^{R-n}w^{\left(R+1\right)k}=1,$ for all other cases of $k=1,\ldots ,n,\;\left(R+1\right)k>R-n$, implying that $D_{w}^{R-n}w^{\left(R+1\right)k}=0$. Hence,

DwR−nawR+1+bn=∑k=0nnkakbn−k10k=bn.

  □

Lemma 2 is almost identical to Lemma 1, with the same outcome but still slightly different from Lemma 1. Furthermore,

(118) $$\Pi \left(s,z,\theta \right)=\frac{1-B_{0}^{N-1}}{1-B_{0}}=\frac{1}{1-sb_{0}\beta_{0}\left(\theta \right)z}\left[1-{\left(sb_{0}\beta_{0}\left(\theta \right)z\right)}^{N-1}\right].$$

In particular,

(119) $$\Pi \left(1,1,0\right)=\frac{1}{1-b_{0}\beta_{0}}\left[1-{\left(b_{0}\beta_{0}\right)}^{N-1}\right].$$

So we close on

(120) $$\begin{array}{c} \psi_{\rho }\left(s,y,z,\theta ;N\right)=Es^{\rho }y^{A_{\rho }}z^{B_{\rho }}e^{-\theta T_{\rho }}\\ =z-z\left[1-s\left[a_{0}z^{N}+b_{0}z\right]\left(\alpha_{0}\left(\theta \right)y+\beta_{0}\left(\theta \right)\right]\right]\Pi \left(s,z,\theta \right) \end{array}$$

after the use of operator $D_{w}^{N-2}$, and summarize it as Theorem 2.

**Theorem** **2.** *In the reliability system (originally set up with four types of shocks: harmless, critical, extreme, and δ-shocks), in which δ-shocks can only be among harmful shocks under the specifications in Section 7, Section 8 and Section 9 and formalized on the traced probability space $\left(\Omega_{0},F\cap \Omega_{0},P_{0}\right)$ $\left[P_{0}=E\left[\left(\cdot \right)1_{\Omega_{0}}\right]\frac{1}{P\left(\Omega_{0}\right)}\right],$ the functional $\psi_{\rho }\left(s,y,z,\theta ;N\right)=Es^{\rho }y^{A_{\rho }}z^{B_{\rho }}e^{-\theta T_{\rho }}$ of the joint transforms of the lifetime $T_{\rho },$ the total shocks count ρ at $T_{\rho },$ the number $A_{\rho }$ of δ-shocks at $T_{\rho },$ and the sum $B_{\rho }$ of all other shock identifiers at $T_{\rho }$, satisfy Formulas $\left(118\right)$ and $\left(120\right)$.*

*Note that $\rho \neq A_{\rho }+B_{\rho }$ as it might be assumed, because $B_{\rho }$ gives the sum of the shock identifiers Us, which assumes values 1 (for a critical shock) and N (for an extreme shock). However, for each $\omega \in \Omega_{0},$ $B_{\rho }\left(\omega \right)$ identifies how many critical and extreme shocks landed by $T_{\rho }\left(\omega \right)$. For example, if $B_{\rho }\left(\omega \right)<N,$ we figure that the number of critical shocks was exactly $B_{\rho }\left(\omega \right),$ with no extreme shock included and with one δ-shock at $T_{\rho }\left(\omega \right),$ which turns out to be the only fatal shock. With $B_{\rho }\left(\omega \right)=N,$ the total harmful shocks count is N. Again, we know that no extreme shock hit the system at $T_{\rho }\left(\omega \right),$ because otherwise, $B_{\rho }\left(\omega \right)$ would have been 2N−1 and not N. We just do not know from $B_{\rho }\left(\omega \right)$ alone if the Nth shock was also δ. Finally, with $B_{\rho }>N,$ we know that the fatal shock at $T_{\rho }\left(\omega \right)$ was extreme or extreme and δ combined.*

**Remark** **10.** *While it is obvious that $\psi_{\rho }$ is given in its closed form through Equations (118) and (120), we conclude our claim of analytical tractability by calling on the special case of Example 3, through a single insertion of*

$$\alpha_{0}\left(\theta \right)=\frac{\gamma p}{\gamma p+\theta }\left[1-e^{-\left(\gamma p+\theta \right)\delta }\right]\;\mathrm{and}\;\beta_{0}\left(\theta \right)=\frac{\gamma p}{\gamma p+\theta }e^{-\left(\gamma p+\theta \right)\delta }$$

*in $\left(118\right)$ and (120) as per Formulas (110) and (111).*

## 10. Marginal Distributions and Means

### 10.1. Time-to-Failure

For s=y=z=1, we arrive at

(121) $$Ee^{-\theta T_{\rho }}=\psi_{\rho }\left(1,1,1,\theta ;N\right)=1-\left[1-\Gamma \left(\theta \right)\right]\Pi \left(1,1,\theta \right),$$

where

(122) $$\Pi \left(1,1,\theta \right)=\frac{1}{1-b_{0}\beta_{0}\left(\theta \right)}\left[1-{\left(b_{0}\beta_{0}\left(\theta \right)\right)}^{N-1}\right]$$

implying that the analog $E\tau_{\rho }$ of (66) reads

(123) $$ET_{\rho }=\frac{1}{\gamma p}\Pi \left(1,1,0\right)=\frac{1}{\gamma p}\frac{1}{1-b_{0}\beta_{0}}\left[1-{\left(b_{0}\beta_{0}\right)}^{N-1}\right].$$

Here, $\frac{1}{\gamma p}=ET_{1}=E\tau_{\eta }$ as per (98).

As in Model 1, because $b_{0}\beta_{0}<1,$ $ET_{\rho }$ is monotone increasing in *N*, converging to $\frac{1}{\gamma p}\frac{1}{1-b_{0}\beta_{0}}$ as N→∞. (See Remark 4 regarding N=∞.) The minimum value of $ET_{\rho }$ is reached at N=2 $\left(\mathrm{recall}\;\mathrm{that}\;N\geq 2\right)$ and it equals to $\frac{1}{\gamma p}$. Furthermore, with N=2,

$$Ee^{-\theta T_{\rho }}=\psi_{\rho }\left(1,1,1,\theta ;2\right)=1-\left[1-\Gamma \left(\theta \right)\right]=\Gamma \left(\theta \right)=\frac{p\gamma \left(\theta \right)}{1-q\gamma \left(\theta \right)}.$$

Here, the system fails regardless of whatever shock (critical, extreme, or δ) strikes it.

### 10.2. δ-Shocks Count

From

$$\begin{array}{c} \psi_{\rho }\left(s,y,z,\theta ;N\right)=Es^{\rho }y^{A_{\rho }}z^{B_{\rho }}e^{-\theta T_{\rho }}\\ \;\;\;\;=z-z\left[1-s\left[a_{0}z^{N}+b_{0}z\right]\left(\alpha_{0}\left(\theta \right)y+\beta_{0}\left(\theta \right)\right]\right]\Pi \left(s,z,\theta \right), \end{array}$$

with s=z=1 and θ=0,

(124) $$\begin{array}{c} \psi_{\rho }\left(1,y,1,0;N\right)=Ey^{A_{\rho }}=1-\left[1-(\alpha_{0}y+\beta_{0}]\right]\Pi \left(1,1,0\right)\\ =1-\left[1-(\alpha_{0}y+\beta_{0}]\right]\frac{1}{1-b_{0}\beta_{0}}\left[1-{\left(b_{0}\beta_{0}\right)}^{N-1}\right]\\ =1-\left[\Pi \left(1,1,0\right)-\Pi \left(1,1,0\right)\left(\alpha_{0}y+\beta_{0}\right)\right]=1-\Pi \left(1,1,0\right)+\Pi \left(1,1,0\right)\beta_{0}+\alpha_{0}\Pi \left(1,1,0\right)y\\ =1-\alpha_{0}\Pi \left(1,1,0\right)+\alpha_{0}\Pi \left(1,1,0\right)y \end{array}$$

implying that $A_{\rho }$ is a Bernoulli r.v. with parameter $\alpha_{0}\Pi \left(1,1,0\right)\;,$ where

(125) $$EA_{\rho }=\alpha_{0}\Pi \left(1,1,0\right)=\frac{\alpha_{0}}{1-b_{0}\beta_{0}}\left[1-{\left(b_{0}\beta_{0}\right)}^{N-1}\right],\;\mathrm{where}\;\alpha_{0}=P\left\{\tau_{\eta }<\delta \right\}.$$

Thus, $EA_{\rho }$ is monotone-increasing in N, with the smallest value at N=2,

$$EA_{\rho }=\alpha_{0}=P\left\{\tau_{\eta }<\delta \right\}$$

and with the sup$EA_{\rho }=\frac{\alpha_{0}}{1-b_{0}\beta_{0}}$. Using straightforward arguments, we can show that for N<∞, the values of $EA_{\rho }=\alpha_{0}\Pi \left(1,1,0\right)=\frac{\alpha_{0}}{1-b_{0}\beta_{0}}<1$. Indeed,

$$\frac{\alpha_{0}}{1-b_{0}\beta_{0}}\;*\;1\;\;\;\Leftrightarrow \;\alpha_{0}\;*\;a_{0}+b_{0}\alpha_{0}\;\;\Leftrightarrow \;\alpha_{0}-b_{0}\alpha_{0}\;*\;a_{0}\;\;\Leftrightarrow \alpha_{0}a_{0}\;*\;a_{0}\Leftrightarrow \;\alpha_{0}\;*\;1.$$

Thus, ∗ is ≤ or rather <, implying that $0<\frac{\alpha_{0}}{1-b_{0}\beta_{0}}<1$. Thus, sup$EA_{\rho }=\alpha_{0}\Pi \left(1,1,0\right)$ is less than 1, unless $\alpha_{0}=1$. With N<∞, however, $EA_{\rho }<1$ even if $\alpha_{0}=1$.

In a nutshell,

$$\alpha_{0}\leq EA_{\rho }<\frac{\alpha_{0}}{1-b_{0}\beta_{0}}$$

implying that the mean δ-shock count lies in $\left[\alpha_{0},\frac{\alpha_{0}}{1-b_{0}\beta_{0}}\right)$. Thus, when N=2, the second shock is a δ-shock with mean value $\alpha_{0}$. Consequently, with N=2,

$$Ey^{A_{\rho }}=\alpha_{0}y+\beta_{0},$$

that is, the marginal $A_{\rho }∼V,$ with

$$\alpha_{0}=P\left\{V=1\right\}=P\left\{\tau_{\eta }<\delta \right\}=EV\;\mathrm{and}\;\beta_{0}=P\left\{V=0\right\}=P\left\{\tau_{\eta }\geq \delta \right\}.$$

Since the system is observed at $T_{0}$ with a prior critical shock$,\;\beta_{0}$ is the probability that a shock at $T_{1}$ is not δ, and with N=2, a non-δ-shock at $T_{1}$ is *N*th critical or extreme.

Now, with $ET_{\rho }=\frac{1}{\gamma p}\Pi \left(1,1,0\right)=\frac{1}{\gamma p}\frac{1}{1-b_{0}\beta_{0}}\left[1-{\left(b_{0}\beta_{0}\right)}^{N-1}\right],$ we conclude that the mean δ-shock value $EA_{\rho }$ is proportional to $ET_{\rho },$ namely,

(126) $$ET_{\rho }=\frac{1}{\gamma p}\frac{1}{\alpha_{0}}EA_{\rho }.$$

### 10.3. Impact $B_{\rho }$ of Critical/Extreme Shocks

Recall that $B_{\rho }=\sum_{k=0}^{\rho }U_{k}$ is the sum of all shocks identifiers collected by the time-to-failure $T_{\rho }$. It thus is an integer with $2\leq B_{\rho }\leq 2N-1$. It is not equal to the shock’s count, because an extreme shock counts as *N* that is the largest quantity of critical shocks. It nevertheless allows us to identify the number of critical and extreme shocks by $T_{\rho }$ as noted at the end of Section 9.

Formally, if $B_{\rho }>N,$ then the system fails due to an extreme shock alone or on the count of an extreme and δ-shock occurring at the same time. If $B_{\rho }=N,$ then the system accumulated exactly *N* critical shocks by $T_{\rho }$ and it failed on *N*th critical shock that turns fatal or on the count of an *N*th critical and δ-shock combined.

If $B_{\rho }<N,$ then $B_{\rho }$ gives the exact number of all critical shocks landing in the system by $T_{\rho }$ when the system fails, and the last of these shocks at $T_{\rho }$ is δ. One needs to be reminded that for various $\omega \in \Omega_{0},$ $B_{\rho }\left(\omega \right)$ can assume any of those named values, and more accurate information comes from the distribution of $B_{\rho }$ obtainable from the marginal PGF $Ez^{B_{\rho }}$.

From

$$\begin{array}{c} \psi_{\rho }\left(s,y,z,\theta ;N\right)=Es^{\rho }y^{A_{\rho }}z^{B_{\rho }}e^{-\theta T_{\rho }}\\ =z-z\left[1-s\left[a_{0}z^{N}+b_{0}z\right]\left(\alpha_{0}\left(\theta \right)y+\beta_{0}\left(\theta \right)\right]\right]\frac{1}{1-sb_{0}\beta_{0}\left(\theta \right)z}\left[1-{\left(sb_{0}\beta_{0}\left(\theta \right)z\right)}^{N-1}\right], \end{array}$$

for s=y=1,θ=0,

$$\begin{array}{c} Ez^{B_{\rho }}=z-z\left[1-\left[a_{0}z^{N}+b_{0}z\right]\right]\frac{1}{1-b_{0}\beta_{0}z}\left[1-{\left(b_{0}\beta_{0}z\right)}^{N-1}\right] \end{array}$$

and $EB_{\rho }=1+\left(a_{0}N+b_{0}\right)\frac{1}{1-b_{0}\beta_{0}}\left[1-{\left(b_{0}\beta_{0}\right)}^{N-1}\right].$

### 10.4. Total Count of Harmful Shocks (Critical/Extreme/δ-Shocks) until Failure

This applies to r.v. ρ and its marginal pgf:

(127) $$Es^{\rho }=1-\left[1-s]\right]\Pi \left(s,1,0\right)=1-\left(1-s\right)\frac{1}{1-sb_{0}\beta_{0}}\left[1-{\left(sb_{0}\beta_{0}\right)}^{N-1}\right],$$

with the expected number of harmful shocks and δ-shocks combined:

(128) $$E\rho =\Pi \left(1,1,0\right)=\frac{1}{1-b_{0}\beta_{0}}\left[1-{\left(b_{0}\beta_{0}\right)}^{N-1}\right]\;=\frac{1}{\alpha_{0}}EA_{\rho }=\gamma pET_{\rho }.$$

From (127), for N=2, ρ=1 a.s., which comes in agreement with the straightforward argument that a second critical shock turns fatal regardless of whether it is second critical, second critical and δ, extreme, or extreme and δ.

Furthermore, Eρ is monotone-increasing in *N* ranging from 1 (at N=2) to $\frac{1}{1-b_{0}\beta_{0}}$, which the supρ. The PGF $Es^{\rho }$ of ρ runs in *s* pointwise at N=2 at the time of failure, to $1-\frac{1-s}{1-sb_{0}\beta_{0}}=s\frac{1-b_{0}\beta_{0}}{1-sb_{0}\beta_{0}}$ when N→∞, which becomes type 1 geometric with parameter $1-b_{0}\beta_{0}$. Obviously, for a very large N, the competition runs exclusively between the extreme and δ-shocks. (Again, see Remark 4 regarding N=∞.)

### 10.5. Monte Carlo Simulation of the Process

We next render Monte Carlo simulations of the Model 2 stochastic process under some specified special cases and compare empirical means derived above as a demonstration the results match empirical findings. In each case, we assume the times between all (harmless and harmful) shocks are exponential (with parameter γ), and we make numerical assumptions about the parameters, including the parameter of time between shocks γ, the time δ, the probabilities of each failure type ($a_{0}$, $b_{0}$), the δ-shock threshold M=1, and the critical shock threshold *N*.

**Figure 15:**
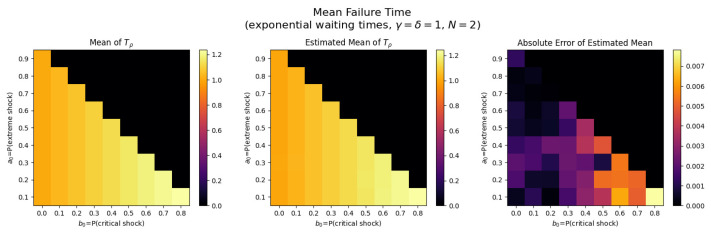
Predicted/estimated mean and absolute error for the failure time $T_{\rho }$.

**Figure 16:**
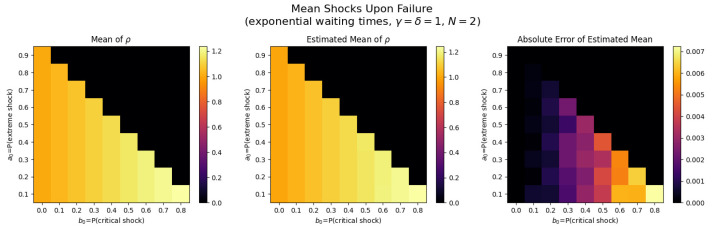
Predicted/estimated mean and absolute error for the shocks ρ.

**Figure 17:**
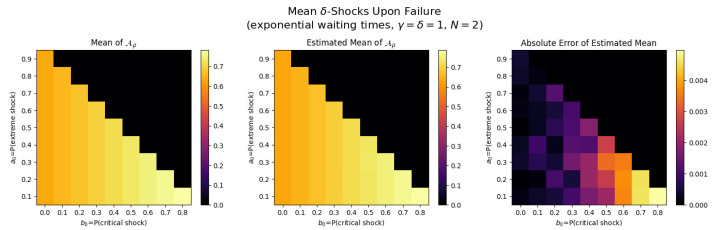
Predicted/estimated mean and absolute error for the δ-shocks $A_{\rho }$.

**Figure 18:**
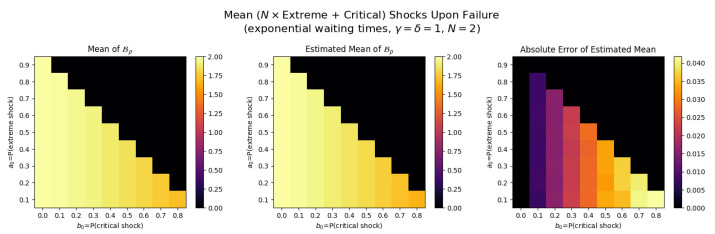
Predicted/estimated mean and absolute error for the (N× Extreme + Critical) shocks $B_{\rho }$.

For the first set of experiments, we set γ=δ=1 and N=2. Figure 15, Figure 16, Figure 17 and Figure 18 below show a comparison of predicted and estimated means of the number of the failure time $\tau_{\rho }$, shocks ρ, δ-shocks $A_{\rho }$ upon failure, and (N× Extreme + Critical) shocks $B_{\rho }$, respectively.

Predicted values come from numerical implementations of (123), (126)–(128) (refer to Appendix A for the Python code). Means are estimated as sample means of each value computed at end of life for a sample of 1,000,000 simulated paths. This experiment is replicated for each $\left(a_{0},b_{0}\right)\in \left\{0.1,0.2,\;\ldots ,0.9\right\}\times \left\{0,0.1,\ldots ,0.8\right\}$.

We display three heat maps: predicted mean, sample mean, and absolute difference for each set of probabilities. In every parameter set tested, the error between the true predicted mean and sample mean is less than 0.04, providing a good validation of the predictions derived above.

We notice $\tau_{\rho }$, ρ, and $A_{\rho }$ have broadly the same pattern: an increase to the critical shock probability $b_{0}$ results in larger means.

In addition, we perform some simulations where δ=1,$\;\left(a_{0},b_{0}\right)=\left(5/9,4/9\right)$, and M=1. Furthermore, we vary the waiting time parameter γ and critical/extreme shock threshold *N* as

(γ,N)∈{0.1,0.2,…,2}×{1,3,5,10}.

**Figure 19:**
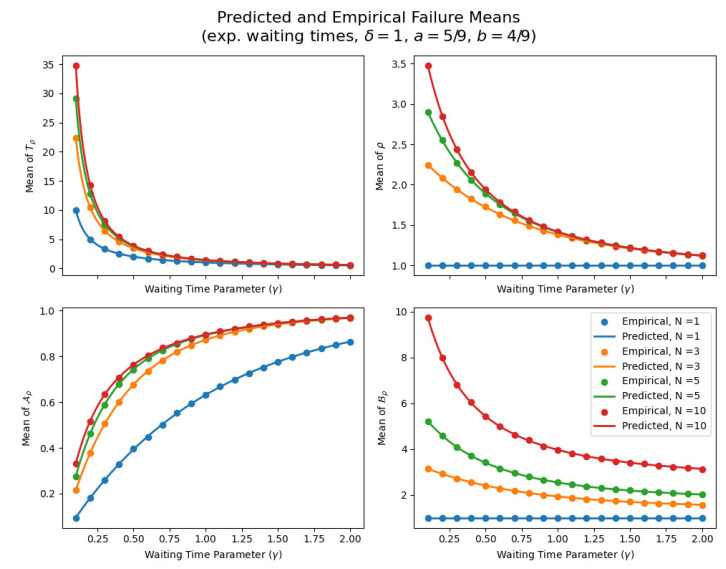
Waiting time parameter (γ) versus predicted/empirical means of ρ, $A_{\rho }$, $B_{\rho }$, and $\tau_{\rho }$.

Sample means here are based on 100,000 simulated paths for every pair (γ,N). Figure 19 below shows the predicted and estimated means of the number of shocks ρ, δ-shocks $A_{\rho }$ upon failure, (N× Extreme + Critical) shocks $B_{\rho }$, and failure time $\tau_{\rho }$. As is seen, the dots (empirical) align precisely with the means derived above and run on a much denser mesh of γ values to form smooth curves, providing additional validation.

We see a good agreement between the true means predicted as the curves and the Monte Carlo simulations as the dots.

## 11. Summary

In this paper, we studied a reliability system subject to random shocks causing different degrees of damages. The shocks enter the system according to a delayed renewal process $\left\{\tau_{n}\right\}$ with respective magnitudes $\left\{W_{n}\right\}$, and they are categorized as harmless, critical, and extreme depending on their strengths relative to two thresholds $0<H_{1}<H_{2}$. We assume that the associated marked renewal process $\sum_{n=0}^{\infty }W_{n}\varepsilon_{\tau_{n}}$ is with position-independent marking; in particular, $W_{n}$s are i.i.d. random variables picked up from an equivalence class $\left[W\right]$. Correspondingly, a shock of magnitude *W* is harmless if $W<H_{1},$ critical if $H_{1}\leq W<H_{2},$ and extreme if $W>H_{2}$.

One of the three events can ruin the system: if there is a single extreme shock; if the system accumulates a total of *N* critical shocks; or if there is a single δ-shock, which is fatal. A δ-shock is defined as an occurrence of two consecutive shocks with a time lag less than some δ. Thus, a pair of two, even harmless, shocks can ruin the system if the time lag between the two is small. Using a common terminology in reliability, there are three competing processes, and the winner is the one that ruins the system first. Understandably, an extreme or *N*th critical shock can occur at the same time as a δ-shock, and thus, two processes may end up sharing the reward.

The objective of this paper was to predict the time-of-failure, as well as the shocks count, including δ-shocks, upon failure. Therefore, of interest was to find the joint distribution of the system’s lifetime and damages incurred upon its ruin. At the same time, we targeted a closed-form functional of such a distribution that was given as an explicit formula in a symbolic form that is reducible to a totally explicit expression once involved input parameters (such as interarrival shock times and their magnitudes in the form of the joint distribution) are specified. We have less interest in working on asymptotic formulas or algorithms.

The results were based on stand-alone Theorem 1, which fitted our system’s settings, although we obtained far more than we needed. More specifically, we considered a marked point process $\left(A,B,\tau \right)=\sum_{k=1}^{\infty }\left(X_{k},Y_{k}\right)\varepsilon_{\tau_{k}}$ describing the evolution of shocks with their magnitudes and respective time lags. Because that process was terminated at $\tau_{\rho }$ (time-to-failure), $\left(A,B,\tau \right)$ was truncated to $\left(A_{\rho },B_{\rho },\tau_{\rho }\right)=\sum_{k=1}^{\rho }\left(X_{k},Y_{k}\right)\varepsilon_{\tau_{k}}$. Theorem 1 established a closed-form expression for the functional

$$\Phi_{\rho }\left(s,y,z,\theta ,u,v,\vartheta ;M,N\right)=Es^{\rho }y^{A_{\rho }}z^{B_{\rho }}u^{A_{\rho -1}}v^{B_{\rho -1}}e^{-\theta \tau_{\rho }-\vartheta \tau_{\rho -1}}$$

and other variants. The formula even allowed us to handle *M* δ-shocks in any order, which we, however, reduced to one (postponing the more general case). A further reduction of $\Phi_{\rho }$ led to functional $\varphi_{\rho }\left(s,y,z,\theta ;N\right)=Es^{\rho }y^{A_{\rho }}z^{B_{\rho }}e^{-\theta \tau_{\rho }}$ (Corollary 1, under M=1). The closed-form claim was fully supported by Example 2.

In Section 5, we discussed marginal distributions and means of time-to-failure $\tau_{\rho },\;\delta$-shock count, the sum of all critical/extreme shocks’ identifiers, and the total shock count, by $\tau_{\rho }$ followed by Monte Carlo simulation of the above process and validation of the results.

Section 6 was dedicated to the functional

$$\Phi_{\mu >\nu }\left(s,y,z,\theta ,s,u,v,\vartheta ;M,N\right)=Es^{\rho }y^{A_{\rho }}z^{B_{\rho }}u^{A_{\rho -1}}v^{B_{\rho -1}}e^{-\theta \tau_{\rho }-\vartheta \tau_{\rho -1}}1_{\mu >\nu }$$

of the general process under the assumption that the system will be ruined by extreme or critical shocks prior to its failure due to δ-shocks (also established in Theorem 1) and its special cases. Of particular interest was the probability $P\left\{\nu <\mu \right\}$ that the failure is due to extreme/critical shocks alone.

In part II of the paper (Section 7, Section 8, Section 9 and Section 10), we introduced a variation of the above model, called Model 2. Namely, in Model 1, we assumed that any two consecutive shocks with a time lag less than δ were deemed fatal regardless of what kind of shocks were involved. This protocol did not exclude incidents with pairs of harmless shocks. In some real-world systems, such an approach seems unwarranted, even though it is universally applied to situations where the magnitudes of shocks are hard or impossible to observe. Yet we decided to offer an alternative model in the event this rule ends up being too rigid. In Model 2, we restricted the use of δ-policy to harmful shocks only. That being said, we bypass harmless shocks and include only critical and extreme shocks with short lags that are now deemed as δ-shocks.

In a nutshell, we singled out those critical or extreme shocks which are consecutive and have time lags smaller than δ. Precisely, they must appear in pairs, and if a shock at some $\tau_{j}$ is critical, we bypassed all harmless shocks at $\tau_{j+1},\tau_{j+2},\ldots$ until the next critical or extreme shock, say, at time $\tau_{j+k},$ comes in a close time proximity from $\tau_{j}$. To proceed further, we singled out the successive epochs $T_{1},T_{2},\ldots ,$ from the sequence $\left\{\tau_{j}\right\}$ of critical and extreme shocks. The interarrival times $T_{1}-T_{0},T_{2}-T_{1},\ldots$ between harmful shocks was easy to find. More challenging was to determine the joint distribution of those times and the identifiers $U_{1},U_{2},\ldots$ of shocks’ magnitudes with identifiers $V_{1},V_{2},\ldots$ of δ-shocks (all binary). We had to use a key fluctuation theorem, previously established. The harmless shocks process was essential in the determination of this joint functional.

After that, we moved to a traced probability subspace where harmless shocks played no role, and we focused on ruin time $T_{\rho }$ of the system on the occasion of extreme, δ-, or *N*th critical shocks or some of their combinations to obtain the joint transform of $T_{\rho }$ and other characteristics at $T_{\rho }$

$$\psi_{\rho }\left(s,y,z,\theta ;N\right)=Es^{\rho }y^{A_{\rho }}z^{B_{\rho }}e^{-\theta T_{\rho }}$$

after using Theorem 1 again, undergoing a similar process as in Model 1, demonstrating again how the new variant can be analyzed through similar methods after making some adjustments. We also confirmed our claim of analytically tractability through various discussions on special cases, examples, and marginal distributions, followed by validation of the results through Monte Carlo simulation.

## Data Availability

Not applicable. We did not collect or use any data.

## Abbreviations and Notation

DCFP	Dependent competing failure processes
$\tau_{1},\tau_{2},\ldots$	Shocks (mixed harmless, critical, extreme) arrival process in Model 1
$\sum_{k=1}^{\infty }\varepsilon_{\tau_{k}}$	The point process of shocks in the form of random measure
$\varepsilon_{a}$	Is Dirac point mass at point a∈R
$W_{1},W_{2},\ldots$	Shocks magnitudes at $\tau_{1},\tau_{2},\ldots$
$\sum_{k=1}^{\infty }W_{k}\varepsilon_{\tau_{k}}$	Shocks process as marked random measure with $\sum_{k=1}^{\infty }\varepsilon_{\tau_{k}}$ as the associated support counting measure
$H_{1},H_{2}$	Shocks’ thresholds
$H_{1}\geq W$	Harmless shock
$H_{1}<W\leq H_{2}$	Critical shock
$H_{2}<W$	Extreme shock
fatal shock	*N*th critical or extreme shock
δ-shock	When $\tau_{i+1}-\tau_{i}<\delta$
r.v.	Random variable
i.i.d.	Independent and identically distributed
$\left[W\right]$	Equivalence class of all r.v.s that are stochastically equivalent to r.v. *W*
$\left(\Omega ,F,P\right)$	Probability space
$E_{1}$	$\left\{W\leq H_{1}\right\}$
$E_{2}$	$\left\{H_{1}<W\leq H_{2}\right\}$
$E_{3}$	$\left\{H_{2}<W\right\}$
$1_{A}$	Indicator function parametrized by an event *A*
shocks identifiers	X,Y, and later on U,V
*Y*	$1_{E_{2}}+N1_{E_{3}}$
$\left\{a,b,c\right\}$	Probability distribution of r.v. *Y*
PGF	Probability generating function
$a\left(z\right)$	$Ez^{Y}=az^{N}+bz+c$ PGF of r.v. *Y*
$\sum_{k=1}^{\infty }Y_{k}\varepsilon_{\tau_{k}}$	Auxiliary point process marked by the sequence $\left\{Y_{k}\right\}$ associated with sequence $\left\{W_{k}\right\}$ of shocks’ magnitudes
$B_{n}$	$\sum_{k=1}^{n}Y_{k},\;n=1,2,\ldots$
ν	Min$\left\{n:B_{n}\geq N\right\}$ nominal count of harmful shocks not counting δ-shocks on system’s failure
$X_{i}$	$1_{\left\{\left|\left(\tau_{i-1},\tau_{i}\right)\right|<\delta \right\}},i=1,2,\ldots ,\tau_{0}=0,$ sequence of i.i.d. Bernoulli r.v.s counting δ-shocks
$\left|A\right|$	Lebesgue measure of Borel set *A*
Δ	An r.v. stochastically equivalent to r.v.s $\left|\left(\tau_{i-1},\tau_{i}\right)\right|,i=1,2,\ldots ,\tau_{0}=0$
α	$P\left\{\Delta <\delta \right\}$
LST	Laplace–Stieltjes transform
$\gamma \left(y,z,\theta \right)$	$Ey^{X}z^{Y}e^{-\theta \Delta }$ the joint transform of $\left(X_{i},Y_{i},\Delta_{i}\right),i=1,2,\ldots$
$\gamma_{0}\left(y,z,\theta \right)$	$Ex^{X_{0}}z^{Y_{0}}e^{-\theta \Delta_{0}}$
$g\left(y,\theta \right)$	$Ey^{X}e^{-\theta \Delta }=Ey^{1_{\left\{\Delta <\delta \right\}}}e^{-\theta \Delta }=\alpha \left(\theta \right)y+\beta \left(\theta \right)$ the marginal transform of $\left(X_{i},\Delta_{i}\right),\;i=1,2,\ldots$
$\alpha \left(\theta \right)$	$E1_{\left\{\Delta <\delta \right\}}e^{-\theta \Delta }$
$\beta \left(\theta \right)$	$E1_{\left\{\Delta \geq \delta \right\}}e^{-\theta \Delta }$
α	$\alpha \left(0\right)$
β	$\beta \left(0\right)$
$\gamma \left(\theta \right)$	$Ee^{-\theta \Delta }=g\left(1,\theta \right)=\alpha \left(\theta \right)+\beta \left(\theta \right)$
$\frac{1}{\gamma }$	EΔ
$A_{n}$	$\sum_{k=1}^{n}X_{k},\;n=1,2,\ldots ,$
μ	Min$\left\{m:A_{m}=M\right\}$
ρ	μ∧ν cumulative ruin indexalso total shocks count on failure in Model 1
$\tau_{\rho }$	Time-to-failure or lifetime of the system in Model 1
$\left(\Omega ,F,\left(F_{t}\right),P\right)$	Filtered probability space
$\varepsilon_{a}$	Dirac (unit) point mass at a point *a*
$\left(A,B,\tau \right)$	$\sum_{k=1}^{\infty }\left(X_{k},Y_{k}\right)\varepsilon_{\tau_{k}}$ marked random measure representing the input stream of shocks
$\left(A_{\rho },B_{\rho },\tau_{\rho }\right)$	$\sum_{k=1}^{\rho }\left(X_{k},Y_{k}\right)\varepsilon_{\tau_{k}}$ input stream of shocks consolidated by $\tau_{\rho }$
$A_{\rho }$	δ-shocks count by $\tau_{\rho }$
$B_{\rho }$	Critical/extreme shocks damage by $\tau_{\rho }$
$\Phi_{\rho }(s,y,z,\theta ,u,v,$ ϑ;M,N)	$Es^{\rho }y^{A_{\rho }}z^{B_{\rho }}u^{A_{\rho -1}}v^{B_{\rho -1}}e^{-\theta \tau_{\rho }-\vartheta \tau_{\rho -1}}$ comprehensive information on the system at time-to-failure $\tau_{\rho },$ including ρ (total shock count on failure); $A_{\rho }$ $\left(\leq M\right)$—the total number of δ-shocks; $B_{\rho }\;\;$—the number of critical and fatal shocks combined; and at time $\tau_{\rho -1}$ preceding to failure, as their joint distribution
$\Phi_{\mu >\nu }(s,y,z,\theta ,s,$ u,v,ϑ;M,N)	status of the system in the form of $Es^{\rho }y^{A_{\rho }}z^{B_{\rho }}u^{A_{\rho -1}}v^{B_{\rho -1}}e^{-\theta \tau_{\rho }-\vartheta \tau_{\rho -1}}1_{\mu >\nu }$ on the confined space $\left(\Omega ,F\cap \left\{\nu <\mu \right\},P_{\left\{\nu <\mu \right\}}\right)$ that fails due to *N*th critical or one extreme shock, but not due to δ-shocks
$D_{x}^{k}F\left(x,y\right)$	D-operator defined as $\lim_{x\to 0}\frac{1}{k!}\frac{\partial^{k}}{\partial x^{k}}\;\left[\;\frac{1}{1-x}\;F\left(x,y\right)\right]$
$\varphi_{\rho }\left(s,y,z,\theta ;N\right)$	$\Phi_{\rho }\left(s,y,z,\theta ,1,1,0;1,N\right)=Es^{\rho }y^{A_{\rho }}z^{B_{\rho }}e^{-\theta \tau_{\rho }}$ marginal functional representing the joint probability distribution of $\left(\rho ,A_{\rho },B_{\rho },\tau_{\rho }\right)$ pertaining to the status of the system predicted by the time-to-failure at $\tau_{\rho }$ in Model 1
$T_{j}$	$\tau_{\eta_{j}}=$ min$\left\{\tau_{n}>T_{j-1}:W_{n}>H_{1}\right\},j=1,2,\ldots ,T_{0}=\tau_{0}=0$ in Model 2
$\eta_{j}$	Min$\left\{n>\eta_{j-1}:W_{n}>H_{1}\right\},\;j=1,2,3,\ldots ,\eta_{0}=0$
$\left\{T_{j}\right\}$	Embedded sequence of consecutive harmful shocks in Model 2
$V_{j}$	$1_{\left\{T_{j}-T_{j-1}<\delta \right\}},j=1,2,\ldots$
$U_{j}$	$N1_{\left\{W_{\eta_{j}}>H_{2}\right\}}+1_{\left\{H_{1}<W_{\eta_{j}}\leq H_{2}\right\}}$
$\left(V,U,T\right)$	$\sum_{k=0}^{\infty }\left(V_{j},U_{j}\right)\varepsilon_{T_{k}}\;\;$delayed marked renewal process representing only harmful shocks in Model 2
*V*	$1_{\left\{\tau_{\eta }<\delta \right\}}$
*p*	a+b
$a_{0}$	$\frac{a}{a+b}\;$
$b_{0}$	$\frac{b}{a+b}\;$
$a_{0}\left(z\right)$	$a_{0}z^{N}+b_{0}z=\frac{1}{p}\left[az^{N}+bz\right]=E\left[z^{Y}1_{\Omega_{0}}\right]\frac{1}{P\left(\Omega_{0}\right)}=\frac{1}{P\left(\Omega_{0}\right)}\int_{\Omega_{0}}z^{Y}dP$
$\Omega_{0}$	$\left\{W>H_{1}\right\}=\left\{Y\in \left\{1,N\right\}\right\}=E_{2}\cup E_{3}$
$\Gamma \left(y,z,\theta \right)$	$Ey^{V}z^{U}e^{-\theta \tau_{\eta }}=a_{0}\left(z\right)G\left(y,\theta \right)=\left(a_{0}z^{N}+b_{0}z\right)\left[\alpha_{0}\left(\theta \right)y+\beta_{0}\left(\theta \right)\right]$
$\Gamma \left(\theta \right)$	$\Gamma \left(1,1,\theta \right)=Ee^{-\theta \tau_{\eta }}=Ee^{-\theta T_{1}}$
$G\left(y,\theta \right)$	$Ee^{-\theta \tau_{\eta }}y^{V}=yEe^{-\theta \tau_{\eta }}1_{\left\{\tau_{\eta }<\delta \right\}}+Ee^{-\theta \tau_{\eta }}1_{\left\{\tau_{\eta }\geq \delta \right\}}=\alpha_{0}\left(\theta \right)y+\beta_{0}\left(\theta \right)$
$\alpha_{0}\left(\theta \right)$	$Ee^{-\theta \tau_{\eta }}1_{\left\{\tau_{\eta }<\delta \right\}}$
$\beta_{0}\left(\theta \right)$	$Ee^{-\theta \tau_{\eta }}1_{\left\{\tau_{\eta }\geq \delta \right\}}$
$\alpha_{0}$	$\alpha_{0}\left(0\right)=P\left\{\tau_{\eta }<\delta \right\}$
$\beta_{0}$	$\beta_{0}\left(0\right)=P\left\{\tau_{\eta }\geq \delta \right\}$
$U_{n}$	$\sum_{k=1}^{n}U_{k},\;n=1,2,\ldots$
ζ	Min$\left\{n:U_{n}\geq N\right\}$
χ	Min$\left\{m:A_{m}=\sum_{k=1}^{m}V_{k}=1\right\}$
ρ	χ∧ζ ruin index in Model 2, also total count of harmful shocks (critical/extreme/δ-shocks) until failure in Model 2
$T_{\rho }$	Time-to-failure in Model 2
${\left(V,U,T\right)}_{\rho }$	$\sum_{k=0}^{\rho }\left(V_{j},U_{j}\right)\varepsilon_{T_{k}}$ input stream of harmful shocks consolidated by $T_{\rho }$
$A_{\rho }$	δ-shocks count by $T_{\rho }$
$B_{\rho }$	Impact of critical/extreme shocks in Model 2
$\psi_{\rho }\left(s,y,z,\theta ;N\right)$	$Es^{\rho }y^{A_{\rho }}z^{B_{\rho }}e^{-\theta T_{\rho }}$ marginal functional representing the joint probability distribution pertaining to the status of the system driven by $\left(\rho ,A_{\rho },B_{\rho },T_{\rho }\right)$ predicted at the time-to-failure at $T_{\rho }$ in Model 2

## Appendix A. Numerical Implementations

In this appendix, we provide Python implementations for the special case results derived in Section 5.

First, we have a function for $\Pi \left(1,1,0\right)$ from (62),

$$\Pi \left(1,1,0\right)=\frac{1}{\alpha +a\beta }\left[1-{\left(\frac{b\beta }{1-c\beta }\right)}^{N}\right]$$

for different values of the the waiting time parameter(s) γ, the δ for δ-shocks, the distribution of shock types (*a* and *b* with *c* computed internally), and the waiting time distribution (implemented for exponential and uniform distributions).

def Pi(gamma, delta, a, b, N, waiting_time_dist=’exponential’):

    # compute alpha (exponential waiting time)

    if waiting_time_dist == ’exponential’:

      alpha = 1 - np.exp(-gamma*delta)

    # compute alpha (uniform[gamma[0],gamma[1]])

    if waiting_time_dist == ’uniform’:

      alpha = (delta - gamma[0])/(gamma[1] - gamma[0])

    beta = 1 - alpha

    c = 1 - a - b

    return (1/(alpha+a*beta))*(1 - (b*beta/(1 - c*beta))**N)

The next function uses the Pi function and returns an array containing Eρ, $EB_{\rho }$, $EA_{\rho }$, and $E\tau_{\rho }$ derived in Section 5:

$$E\rho =\Pi \left(1,1,1,0;M,N\right)$$

$$E\tau_{\rho }=\left(E\Delta \right)\Pi \left(1,1,1,0;M,N\right)$$

$$EB_{\rho }=\left(aN+b\right)\Pi \left(1,1,1,0;M,N\right)$$

$$EA_{\rho }=P\left\{\left|\Delta \right|<\delta \right\}\Pi \left(1,1,1,0;M,N\right)$$

def compute_means(gamma, delta, a, b, N, waiting_time_dist=’exponential’):

    # compute alpha (exponential waiting time)

    if waiting_time_dist == ’exponential’:

      alpha = 1 - np.exp(-gamma*delta)

      mean_waiting_time = 1/gamma

    # compute alpha (uniform[gamma[0],gamma[1]])

    if waiting_time_dist == ’uniform’:

      alpha = (delta - gamma[0])/(gamma[1] - gamma[0])

      mean_waiting_time = (gamma[0] + gamma[1])/2

    pi = Pi(gamma, delta, a, b, N)

    mean_failure_time = pi*mean_waiting_time

    mean_failure_index = pi

    mean_extreme_critical_damage = (a*N + b)*pi

    mean_delta_damage = alpha*pi

    return np.array([a, b, mean_failure_index,

               mean_extreme_critical_damage,

               mean_delta_damage,

               mean_failure_time])

These functions are used for predicted values of the figures in Section 5.6.

## Appendix B. Monte Carlo Simulations

In this appendix, we include the Python code used to simulate paths of the full reliability system. All empirical sample means in the diagrams of Section 5.6 are based on running the function below.

Its inputs are the waiting time parameter(s) γ, the δ for δ-shocks, the distribution of shock types (*a* and *b* with *c* computed internally), and the waiting time distribution (implemented for exponential and uniform distributions).

It then simulates the full path to failure, outputting values at failure time including number of shocks (“failure_index”); prefailure and failure time; numbers of extreme, critical, and δ-shocks upon prefailure and failure times; and binary flags for whether the fatal shock is an extreme shock, critical shock, or δ-shock.

def simulate_path(gamma, delta, a, b, N, waiting_time_dist=’exponential’, verbose=False):

    # initialize outputs

    failure_index = 0

    failure_time = 0

    extreme_damage = 0

    critical_damage = 0

    delta_damage = 0

    extreme_failure = False

    critical_failure = False

    delta_failure = False

    rng = np.random.default_rng()

    # compute alpha (exponential waiting time)

    if waiting_time_dist == ’exponential’:

      alpha = 1 - np.exp(-gamma*delta)

    # compute alpha (uniform[gamma[0],gamma[1]])

    if waiting_time_dist == ’uniform’:

      alpha = (delta - gamma[0])/(gamma[1] - gamma[0])

    beta = 1 - alpha

    c = 1 - a - b

    # simulate the process

    while extreme_failure + critical_failure + delta_failure == 0:

      # save prior values

      old_extreme_damage = extreme_damage

      old_critical_damage = critical_damage

      old_delta_damage = delta_damage

      old_failure_time = failure_time

      # compute waiting time before the next shock

      if waiting_time_dist == ’exponential’:

        waiting_time = rng.exponential(1/gamma)

      if waiting_time_dist == ’uniform’:

        waiting_time = rng.uniform(gamma[0], gamma[1])

      # increment failure index

      failure_index += 1

      if verbose: print(f’Shock number {failure_index}’)

      # add to failure_time

      failure_time += waiting_time

      # delta shock

      if waiting_time < delta:

        if verbose: print(’Delta shock!’)

        delta_damage += 1

        delta_failure = True

      # simulate shock

      shock_strength = rng.random()

      # extreme shock

      if shock_strength < a:

        if verbose: print(’Extreme shock!’)

        extreme_damage += 1

        extreme_failure = True

      # critical shock

      elif shock_strength < a + b:

        if verbose: print(’Critical shock!’)

        critical_damage += 1

        if critical_damage == N:

          critical_failure = True

    return np.array([failure_index,

               old_extreme_damage, extreme_damage,

               old_critical_damage, critical_damage,

               old_delta_damage, delta_damage,

               old_failure_time, failure_time,

               extreme_failure, critical_failure, delta_failure])

Empirical sample means for ρ, $A_{\rho }$, and $\tau_{\rho }$ presented in Section 5.6 are found in each case by running this function 1 m times, averaging the first, seventh, and ninth outputs, respectively. Since the simulation outputs extreme shocks and critical shocks separately (unlike the model), we simply compute

critical_damage + N*extreme_damage

before taking the sample mean for $B_{\rho }$.
